# Supplementary figures and images for: Fibroblast hierarchy dynamics during mammary gland morphogenesis and tumorigenesis
Source: EMBO J. 2025 Apr 11;44(11):3266–300. doi: 10.1038/s44318-025-00422-3 (PMC12130467; doi:10.1038/s44318-025-00422-3)

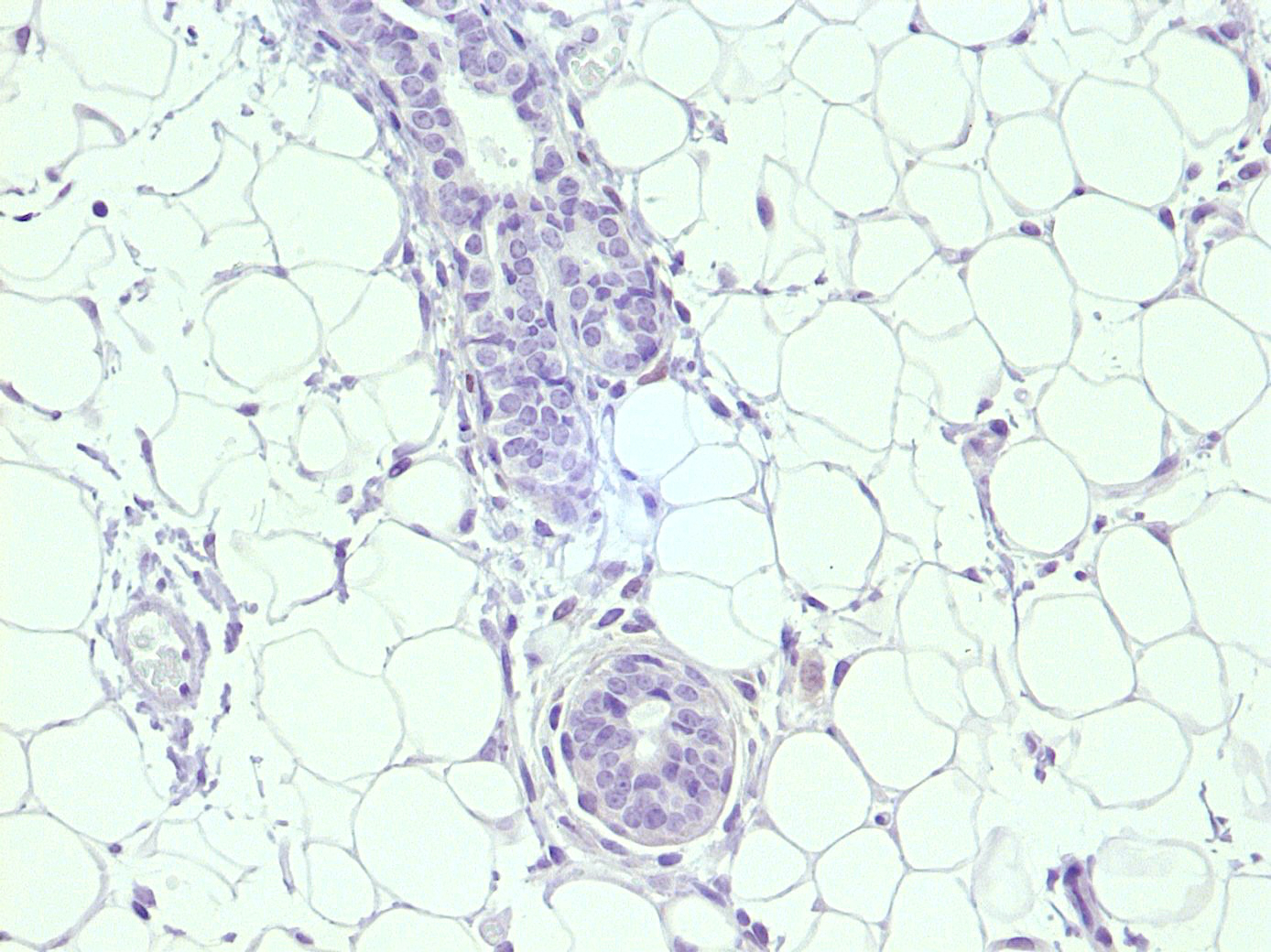

Supplement: Supplementary file 8 — Source data Fig. 3 [file 44318_2025_422_MOESM8_ESM.zip › Figure 3/Fig 3e/Fig 3e CRABP1 _PLACEBO.jpg]

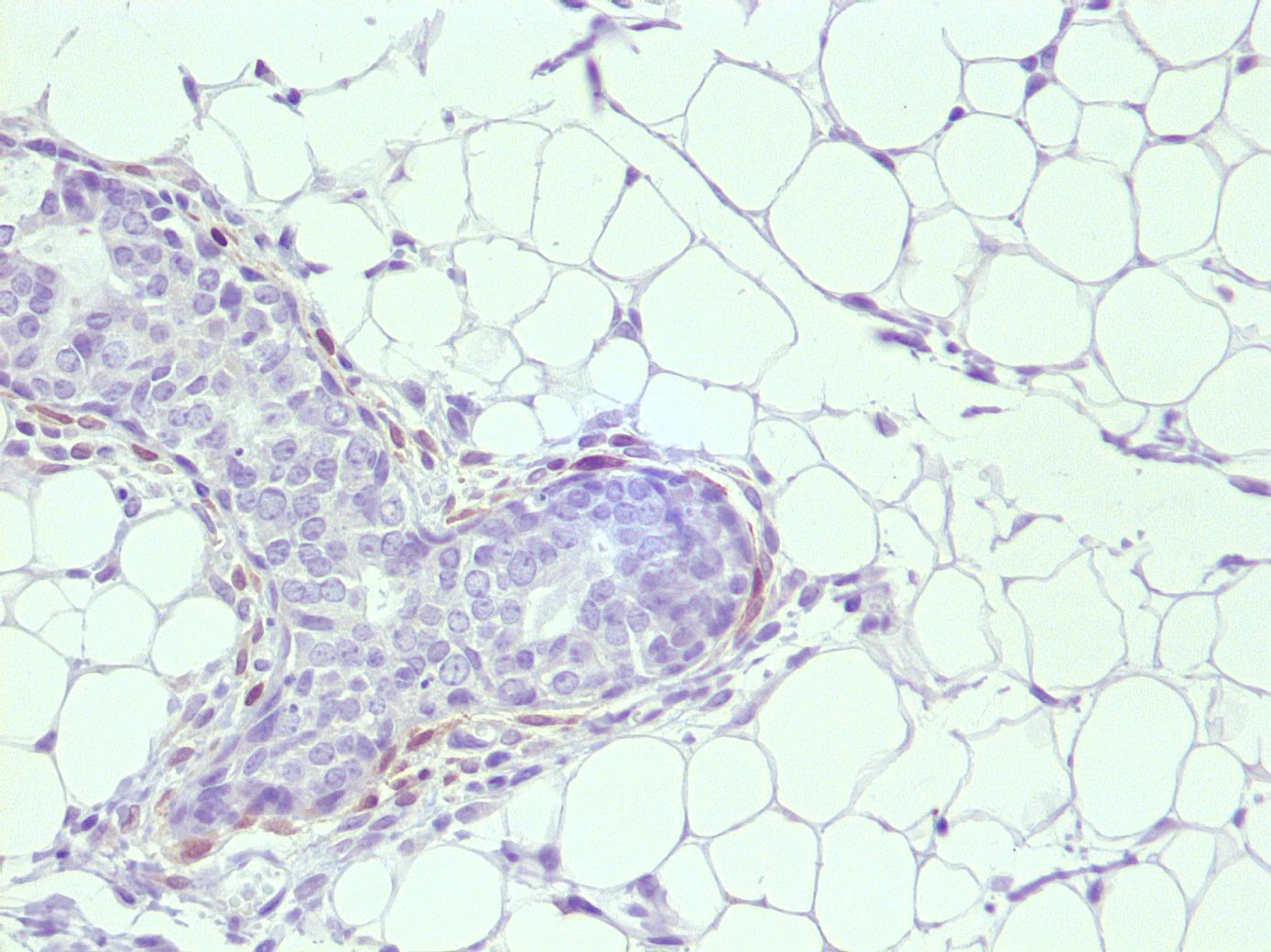

Supplement: Supplementary file 8 — Source data Fig. 3 [file 44318_2025_422_MOESM8_ESM.zip › Figure 3/Fig 3e/Fig 3e CRABP1 MPA.jpg]

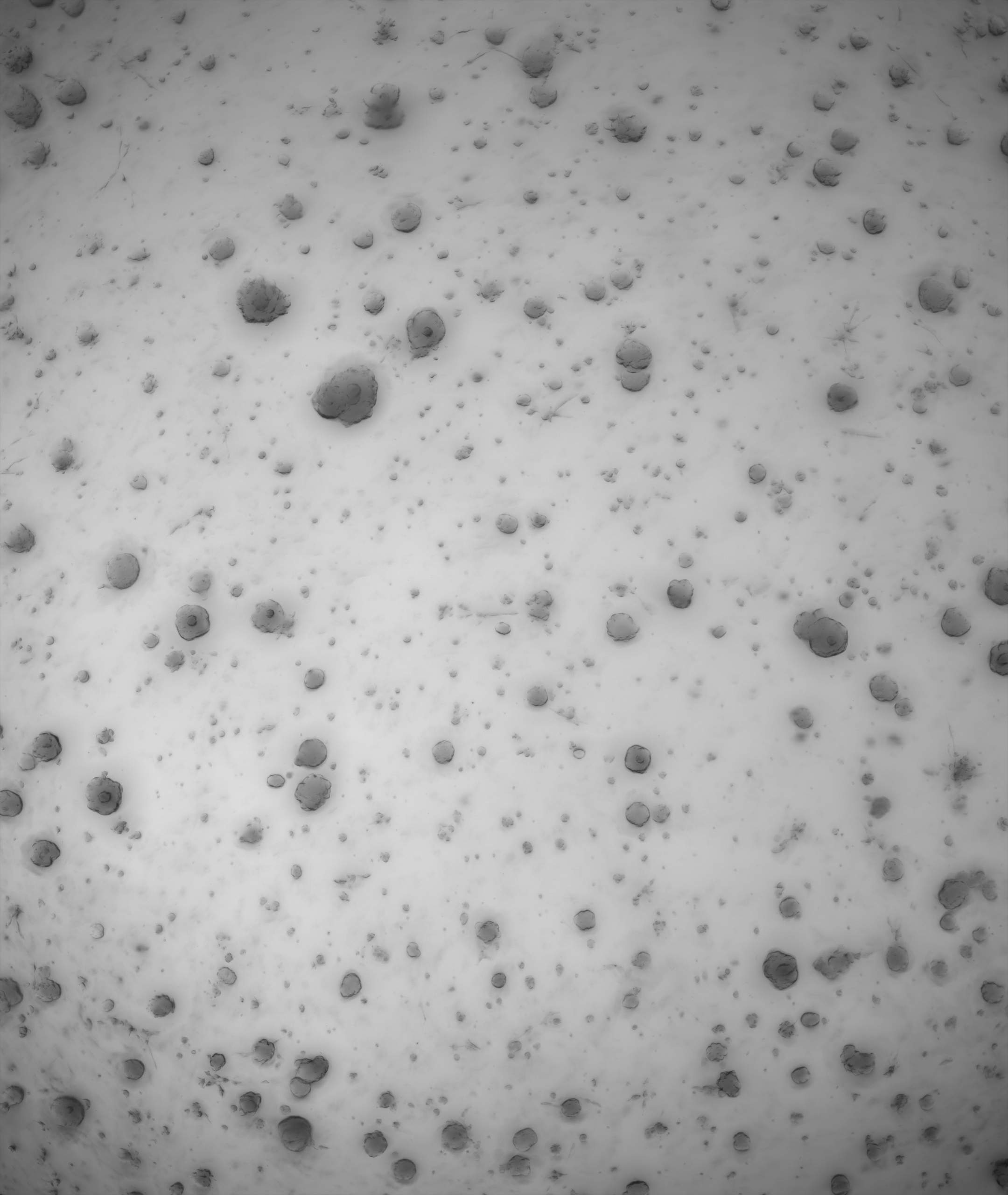

Supplement: Supplementary file 9 — Source data Fig. 5 [file 44318_2025_422_MOESM9_ESM.zip › Figure 5/Fig 5a/Fig 5a_Wnt1_Basal+CAF.tif]

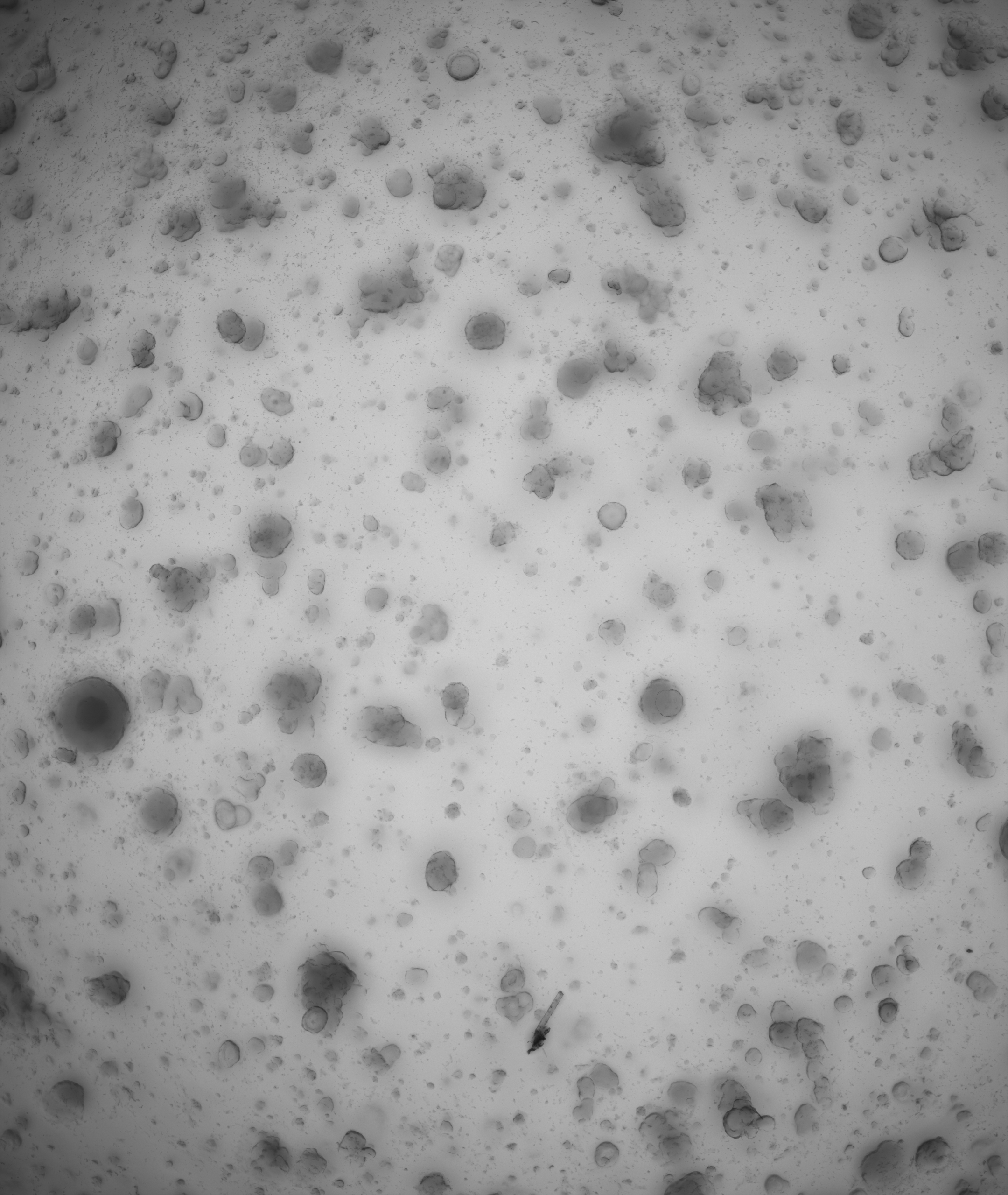

Supplement: Supplementary file 9 — Source data Fig. 5 [file 44318_2025_422_MOESM9_ESM.zip › Figure 5/Fig 5a/Fig 5a_Wnt1_LP+CAF.tif]

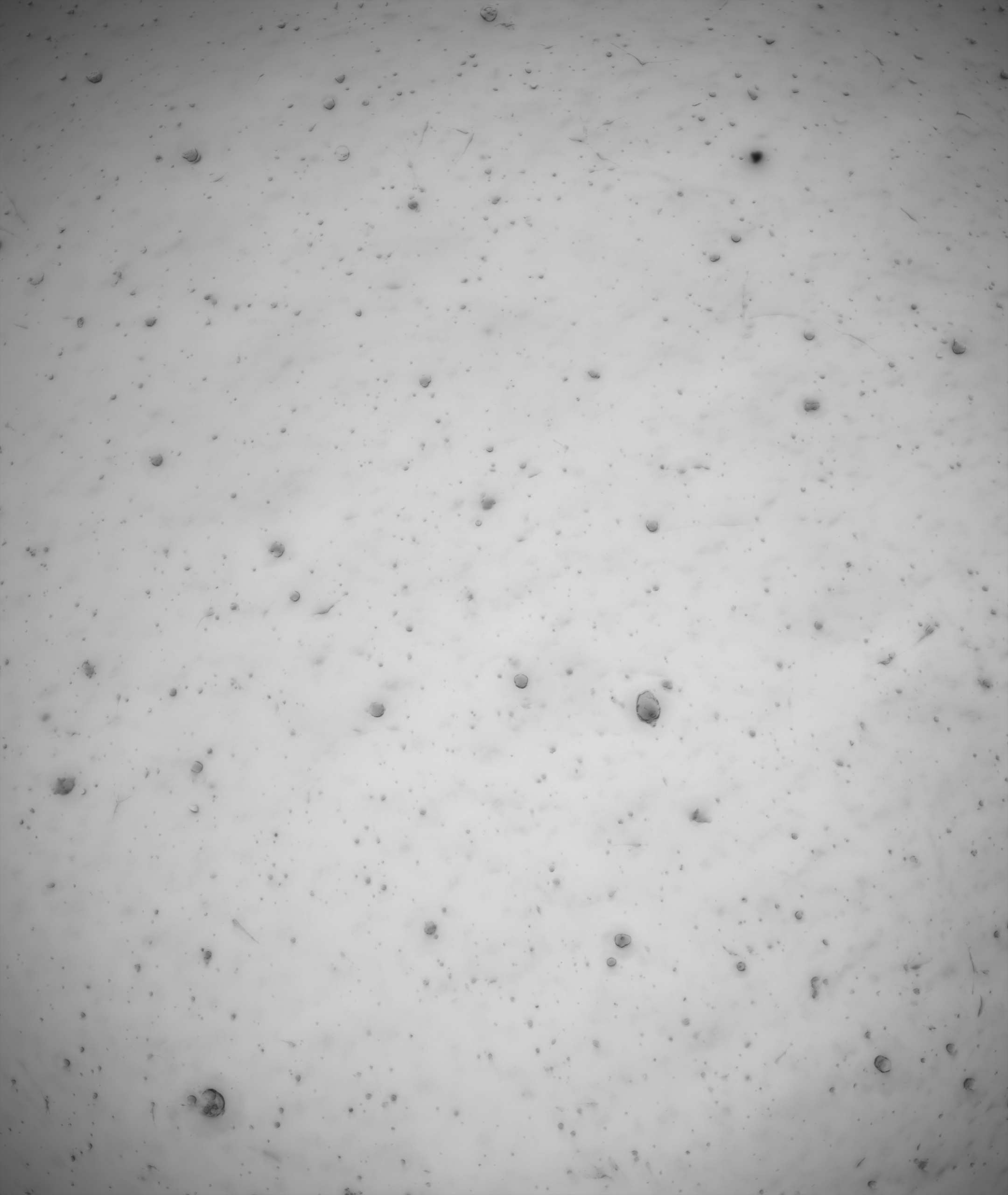

Supplement: Supplementary file 9 — Source data Fig. 5 [file 44318_2025_422_MOESM9_ESM.zip › Figure 5/Fig 5a/Fig 5a_Wnt1_Basal+HAF.tif]

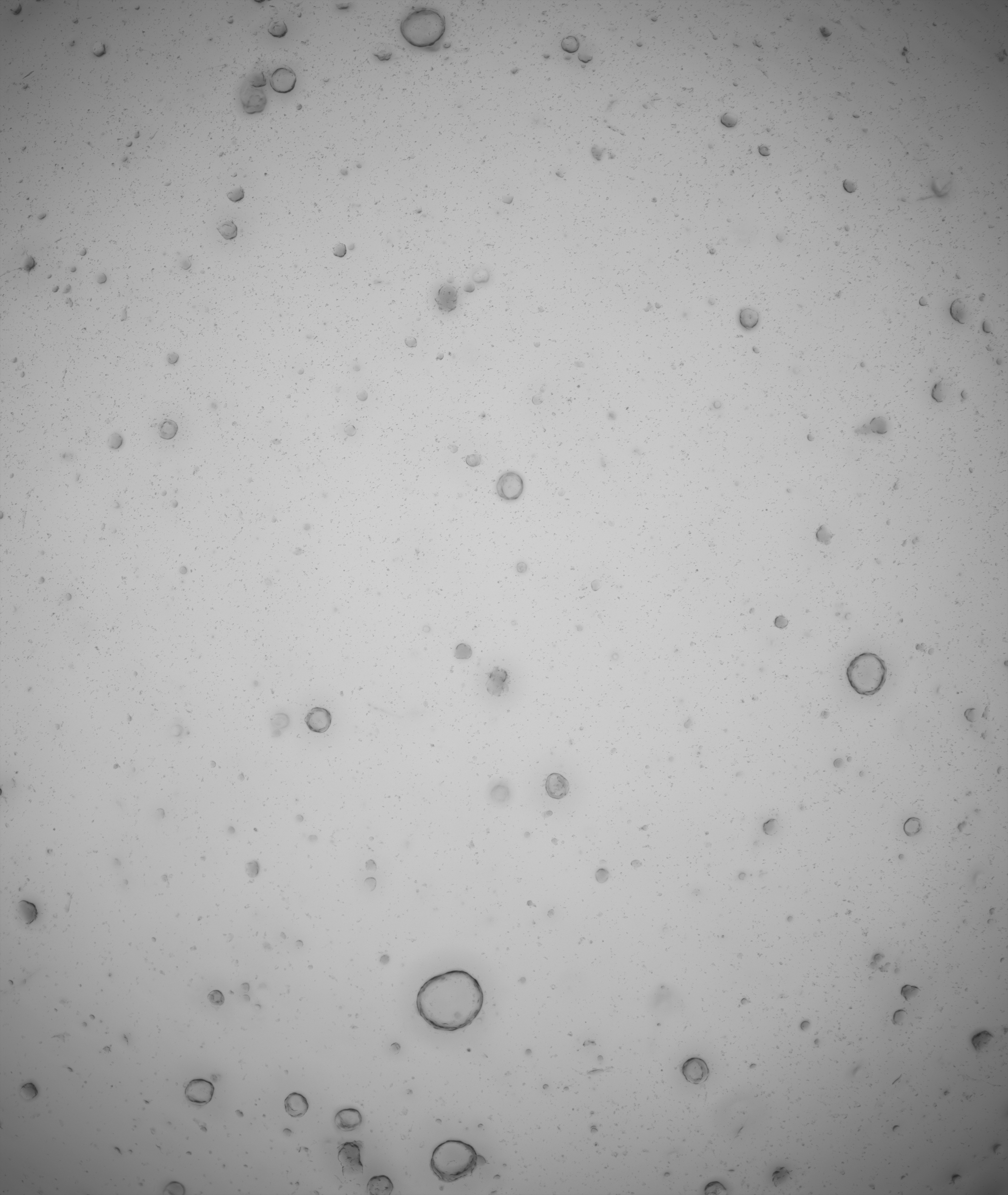

Supplement: Supplementary file 9 — Source data Fig. 5 [file 44318_2025_422_MOESM9_ESM.zip › Figure 5/Fig 5a/Fig 5a_Wnt1_LP+HAF.tif]

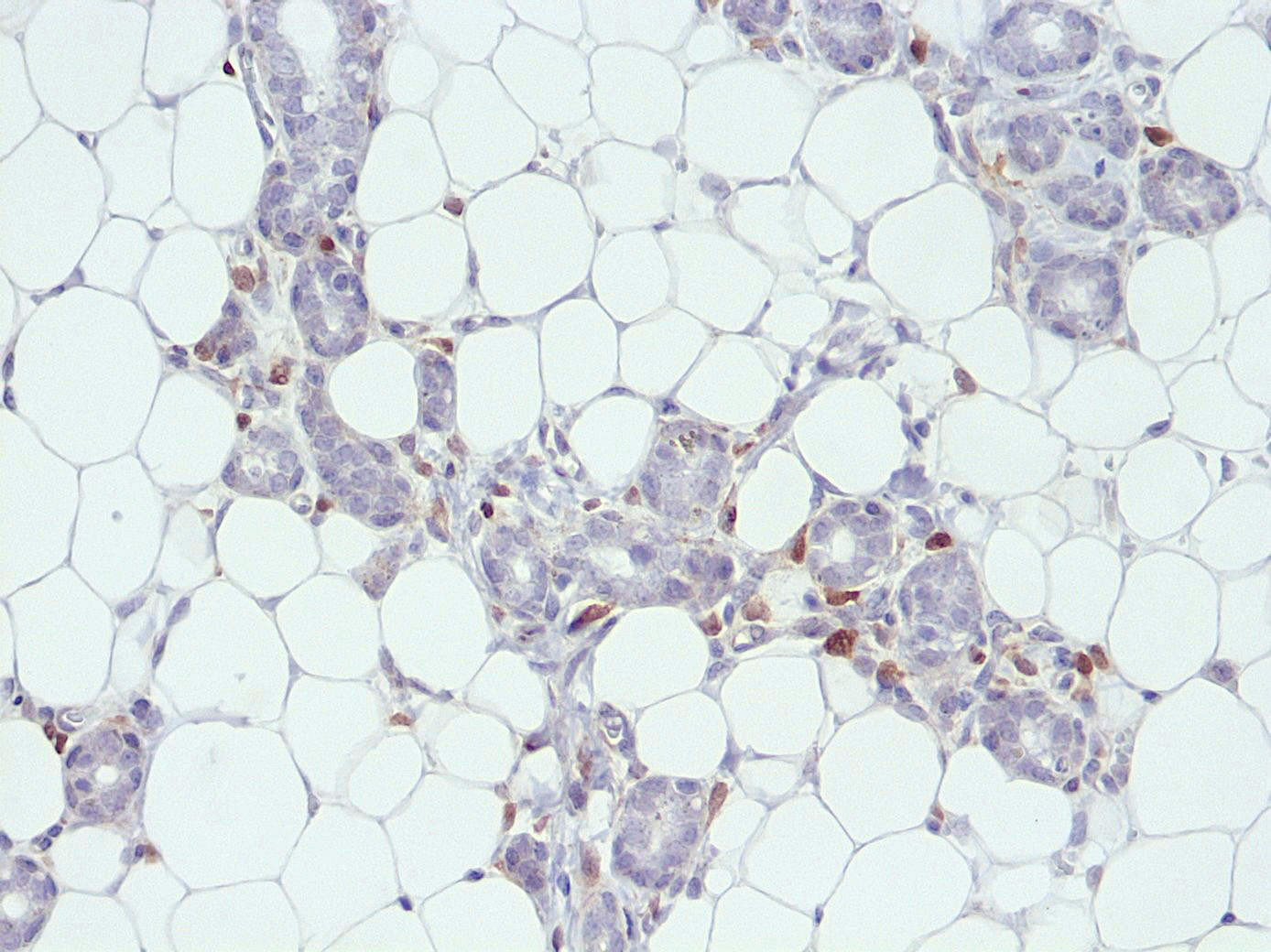

Supplement: Supplementary file 9 — Source data Fig. 5 [file 44318_2025_422_MOESM9_ESM.zip › Figure 5/Fig 5h/Fig 5h CRABP1 hyper.jpg]

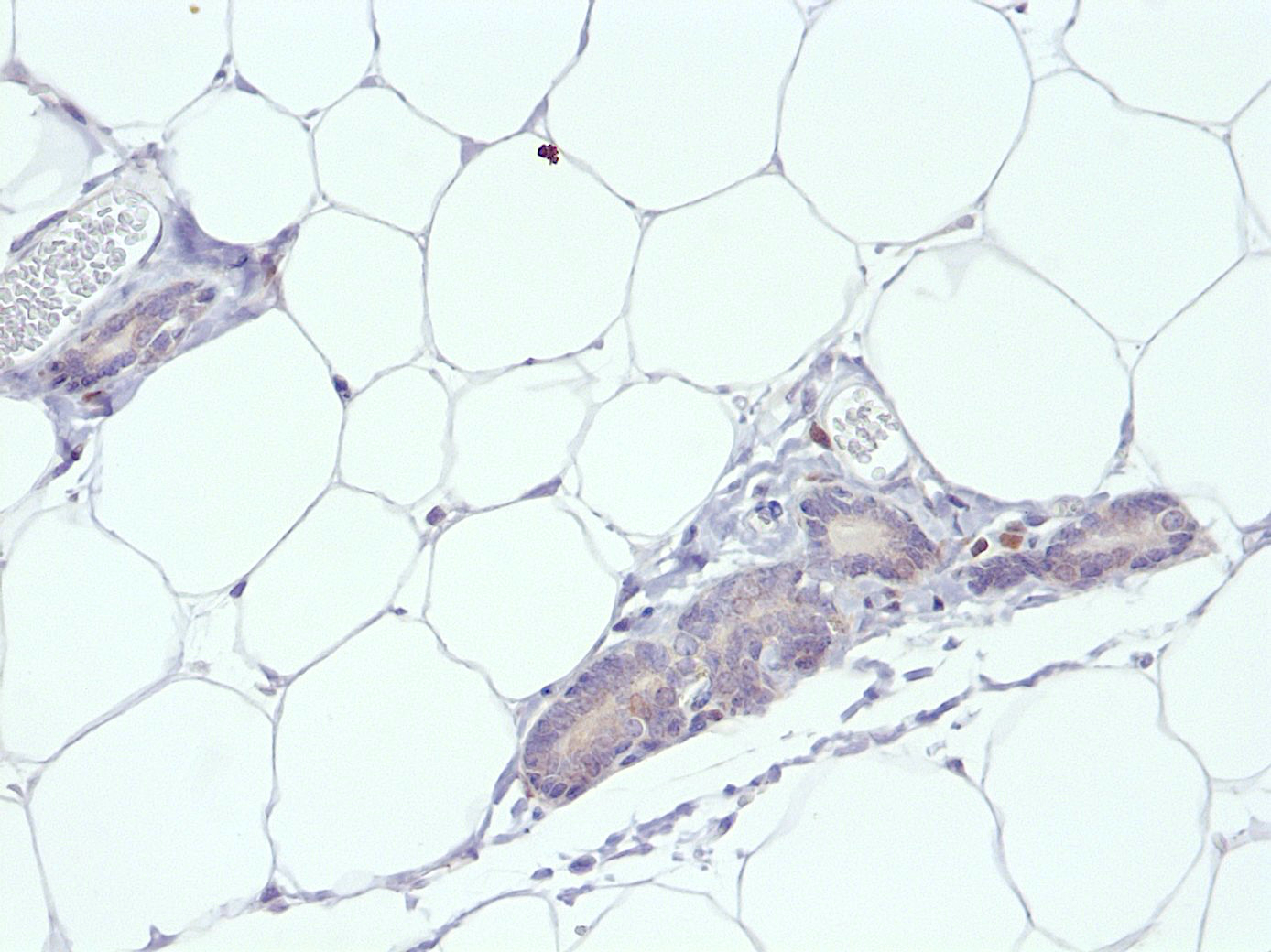

Supplement: Supplementary file 9 — Source data Fig. 5 [file 44318_2025_422_MOESM9_ESM.zip › Figure 5/Fig 5h/Fig 5h CRABP1 ctrl.jpg]

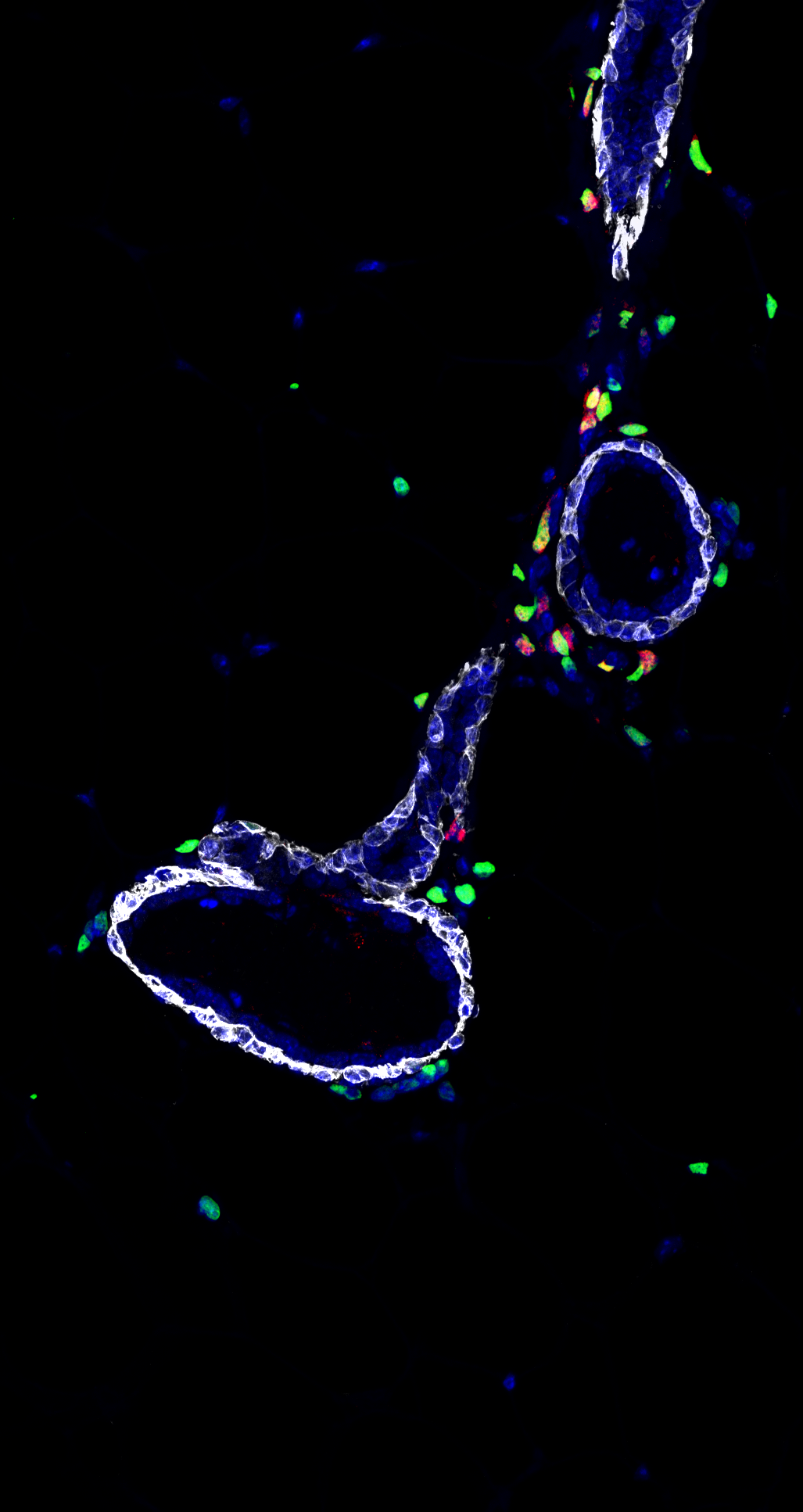

Supplement: Supplementary file 9 — Source data Fig. 5 [file 44318_2025_422_MOESM9_ESM.zip › Figure 5/Fig 5i/Fig 5i MAX_CRABP1 MGhyper.tif]

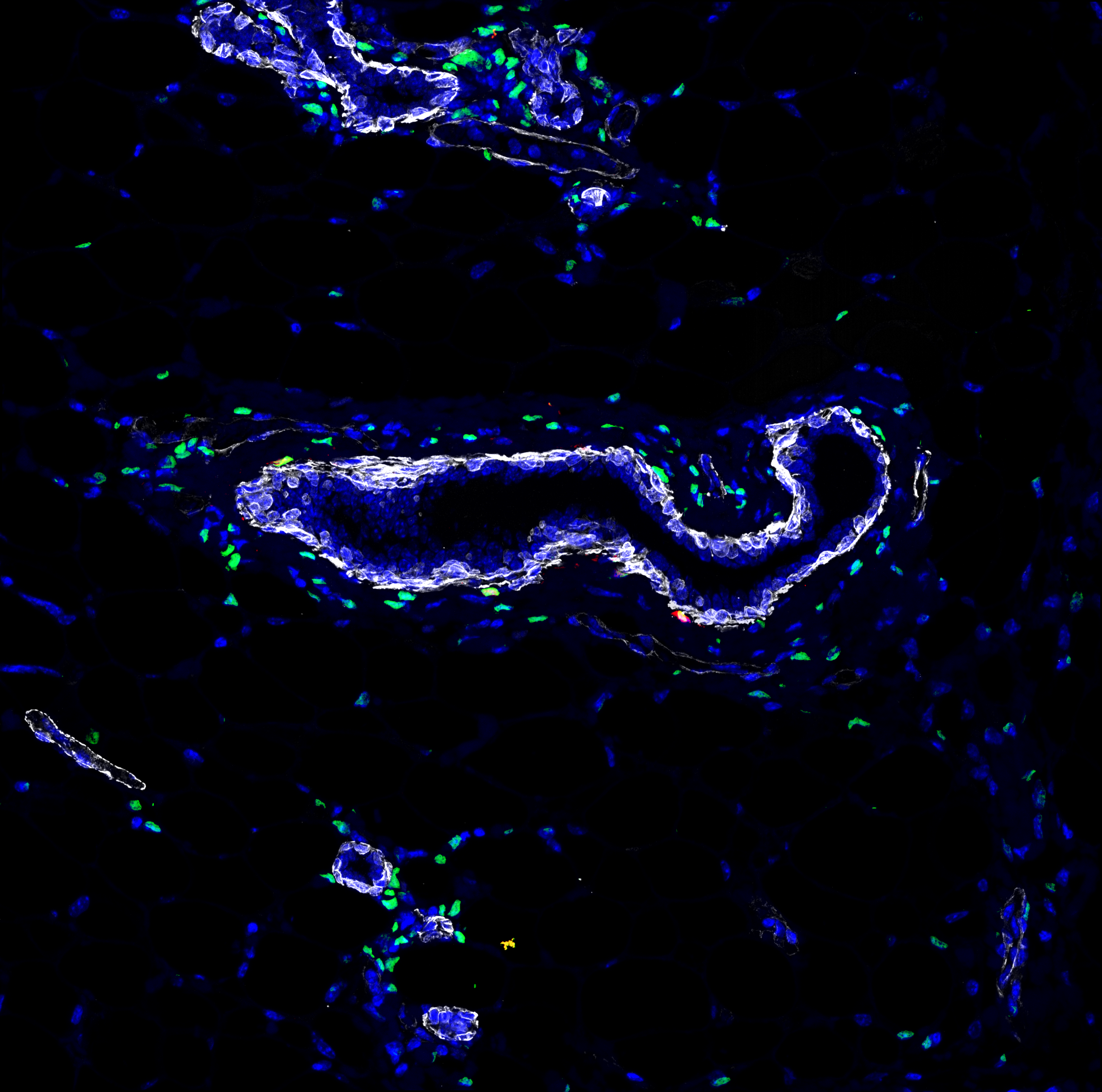

Supplement: Supplementary file 9 — Source data Fig. 5 [file 44318_2025_422_MOESM9_ESM.zip › Figure 5/Fig 5i/Fig 5i MAX_CRABP1 control.tif]

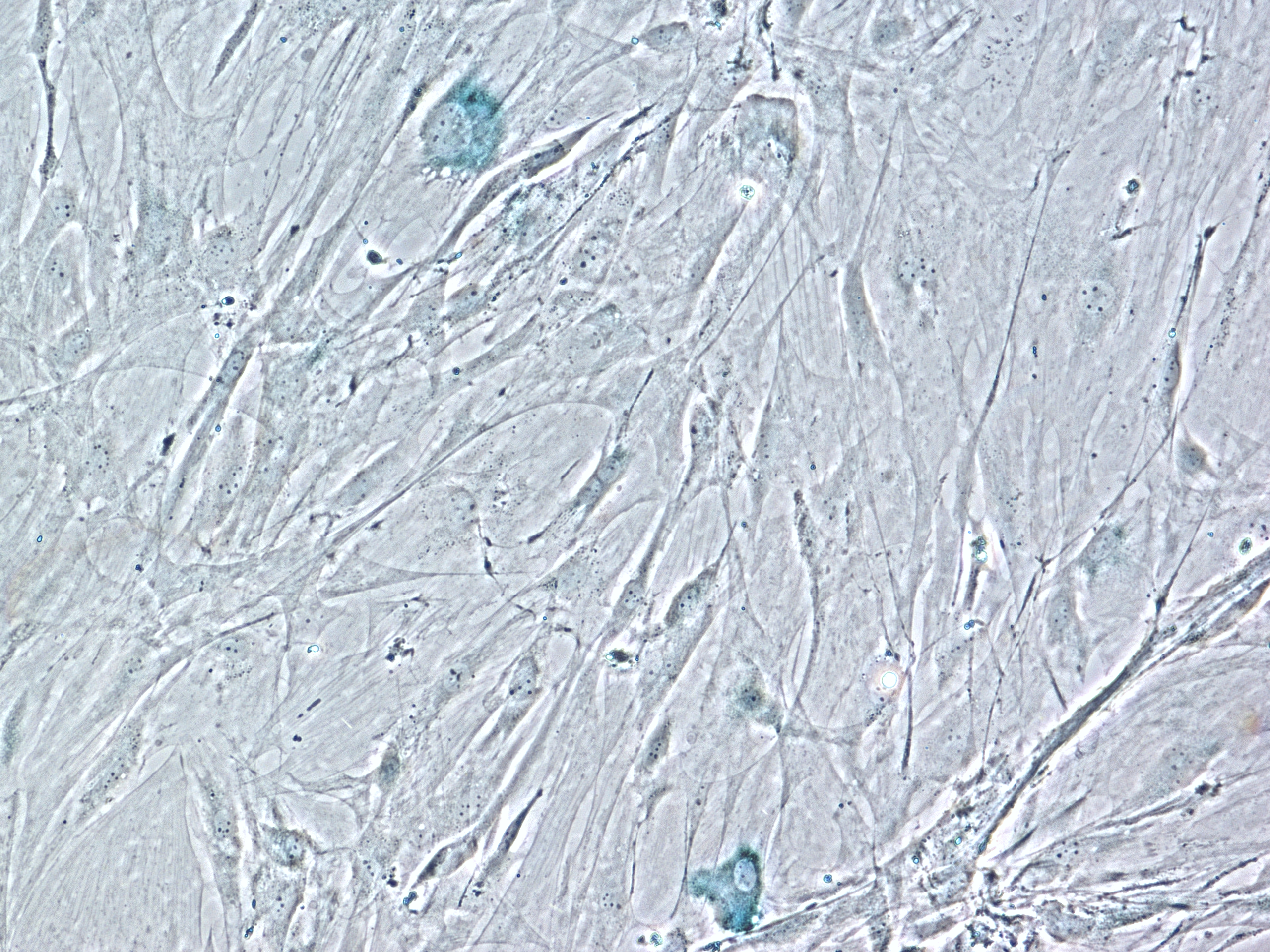

Supplement: Supplementary file 10 — Source data Fig. 6 [file 44318_2025_422_MOESM10_ESM.zip › Figure 6/Fig 6k/Fig 6k_CD34+ vehicle 10X.jpg]

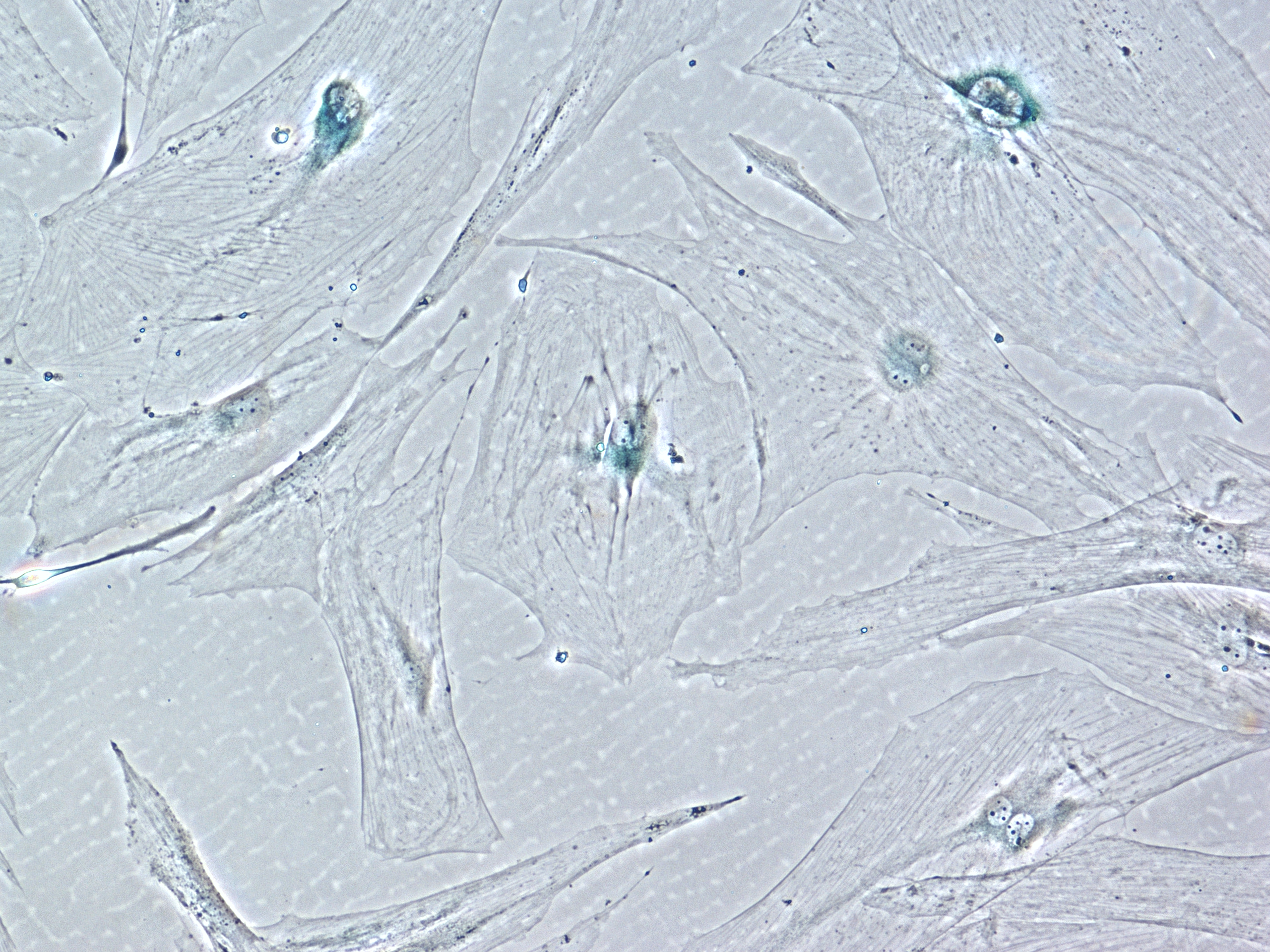

Supplement: Supplementary file 10 — Source data Fig. 6 [file 44318_2025_422_MOESM10_ESM.zip › Figure 6/Fig 6k/Fig 6k_CD34+ etoposide 10X.jpg]

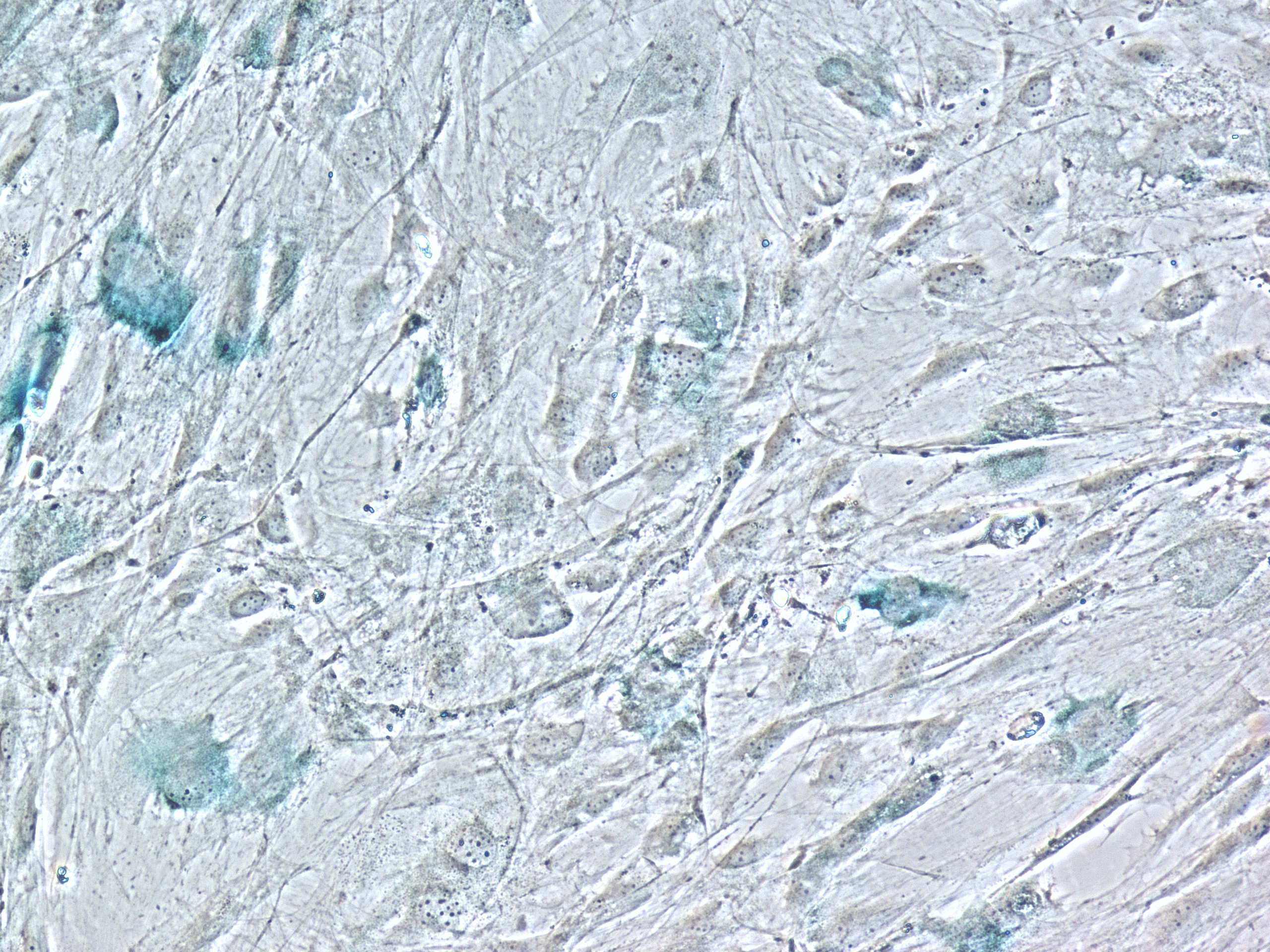

Supplement: Supplementary file 10 — Source data Fig. 6 [file 44318_2025_422_MOESM10_ESM.zip › Figure 6/Fig 6k/Fig 6k_CD34- vehicle 10X.jpg]

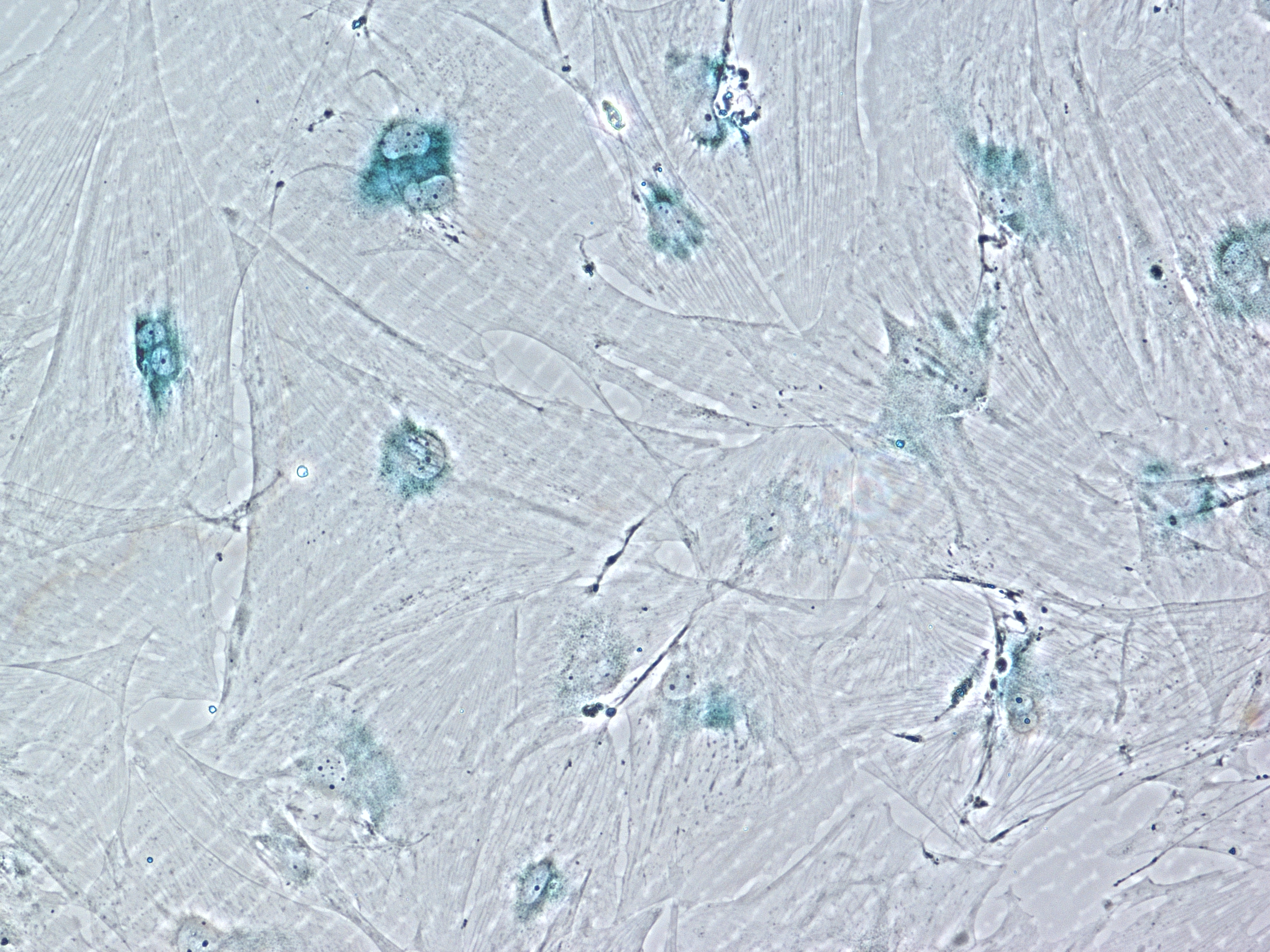

Supplement: Supplementary file 10 — Source data Fig. 6 [file 44318_2025_422_MOESM10_ESM.zip › Figure 6/Fig 6k/Fig 6k_CD34- etoposide 10X.jpg]

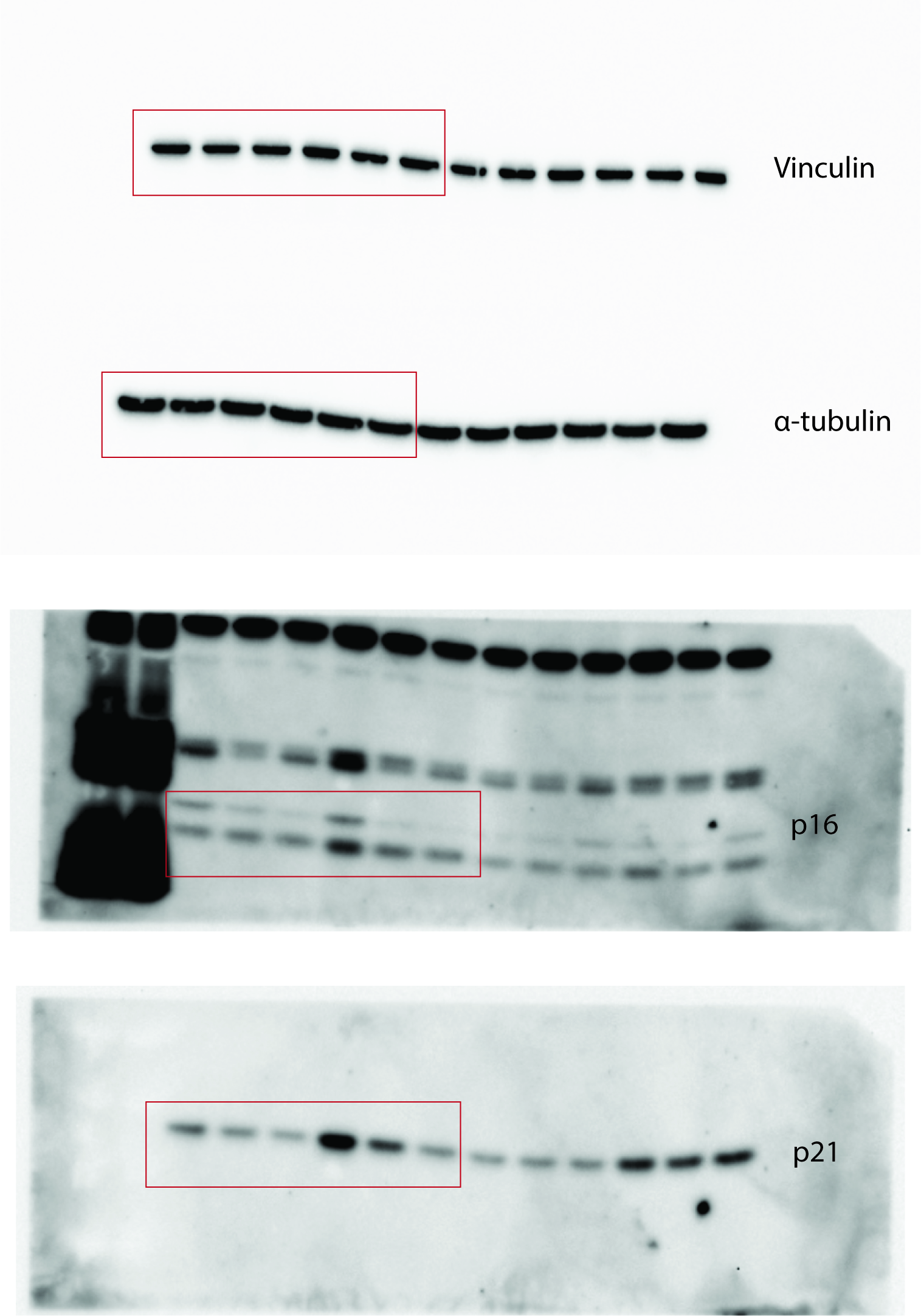

Supplement: Supplementary file 11 — Source data Fig. 7 [file 44318_2025_422_MOESM11_ESM.zip › Figure 7/Fig 7e_WB.tif]

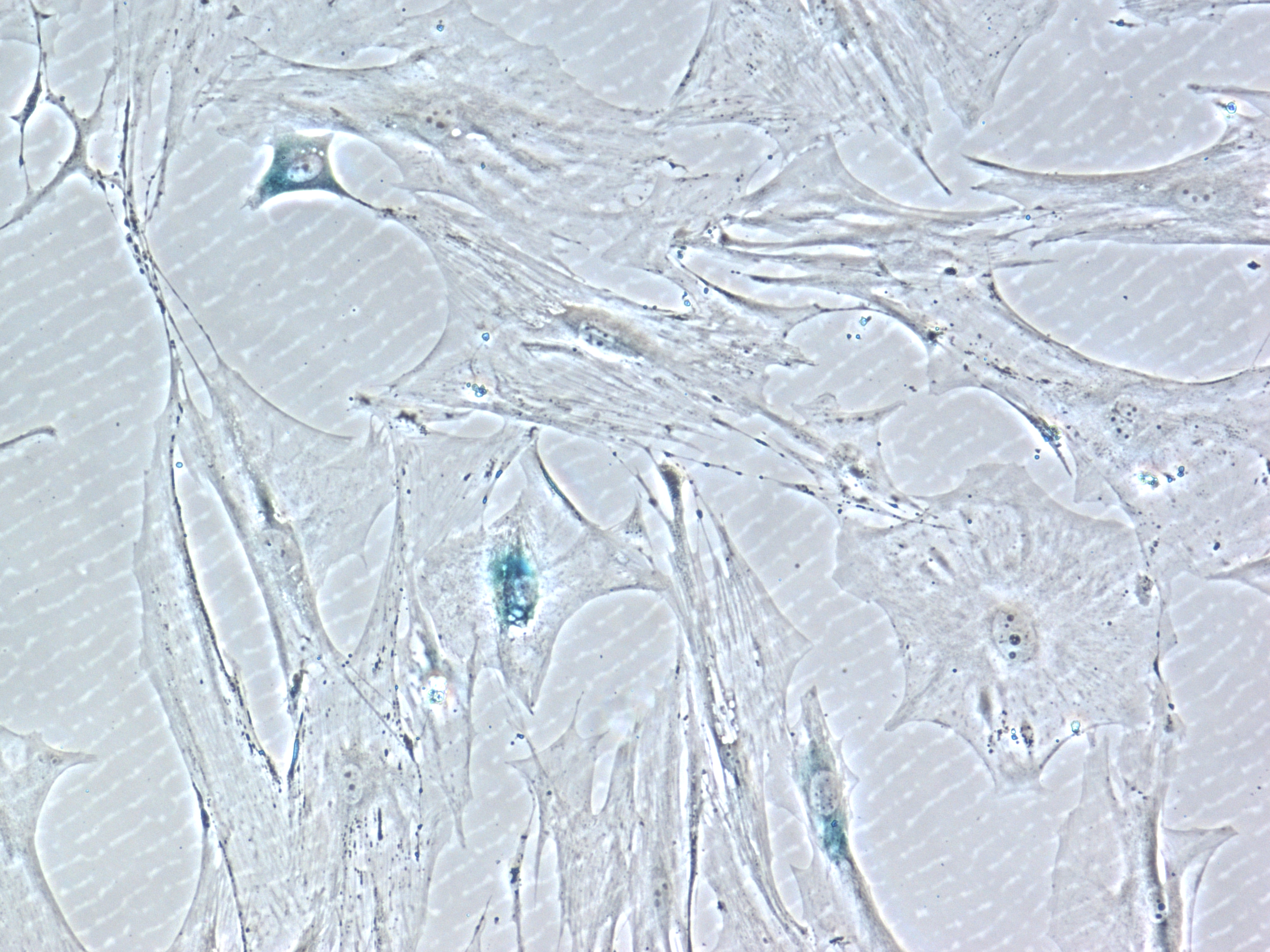

Supplement: Supplementary file 11 — Source data Fig. 7 [file 44318_2025_422_MOESM11_ESM.zip › Figure 7/Fig 7c/Fig 7c_sgNT 1799 etoposide_10X.jpg]

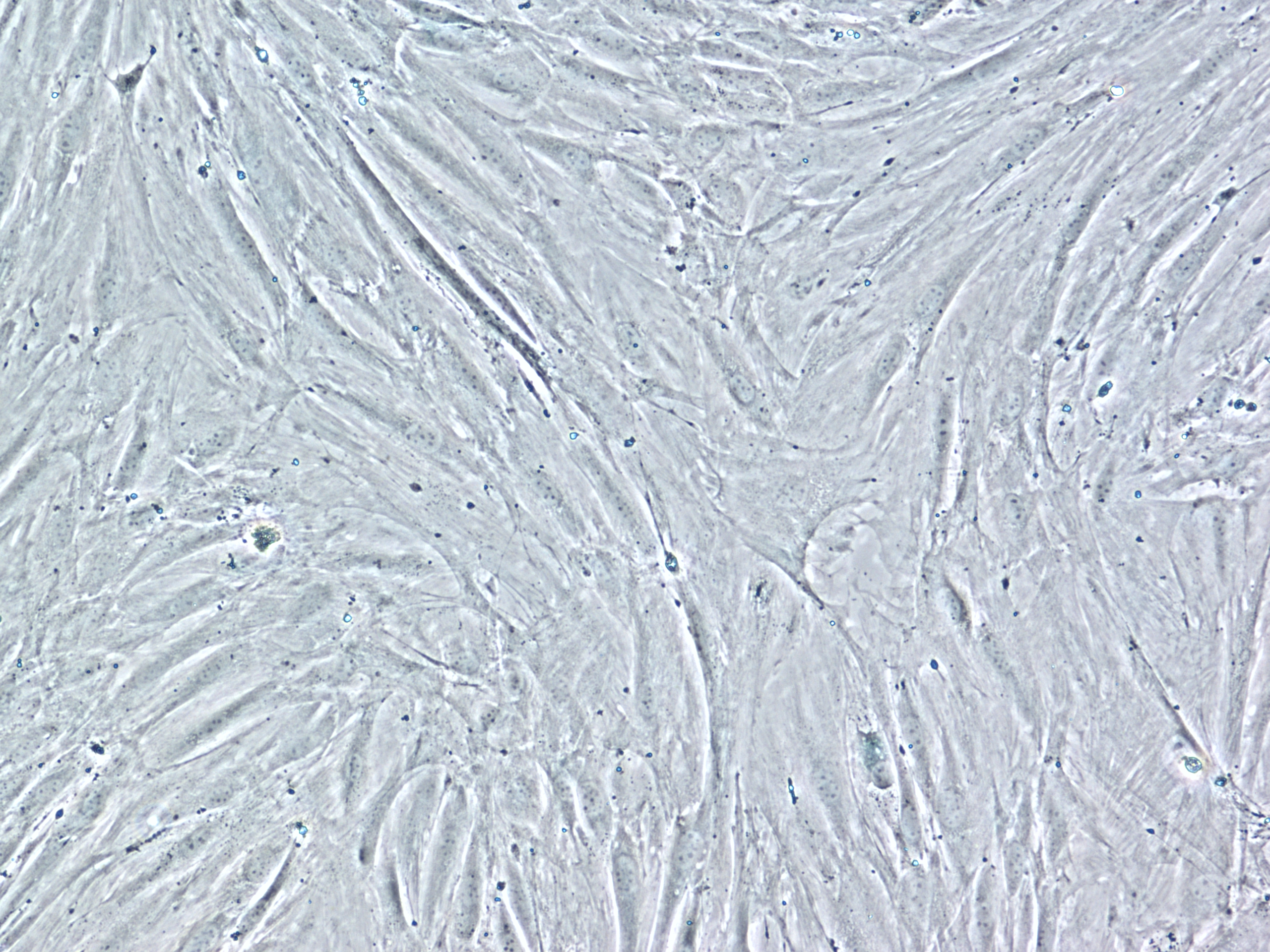

Supplement: Supplementary file 11 — Source data Fig. 7 [file 44318_2025_422_MOESM11_ESM.zip › Figure 7/Fig 7c/Fig 7c_sgWnt9a 2194 vehicle_10X.jpg]

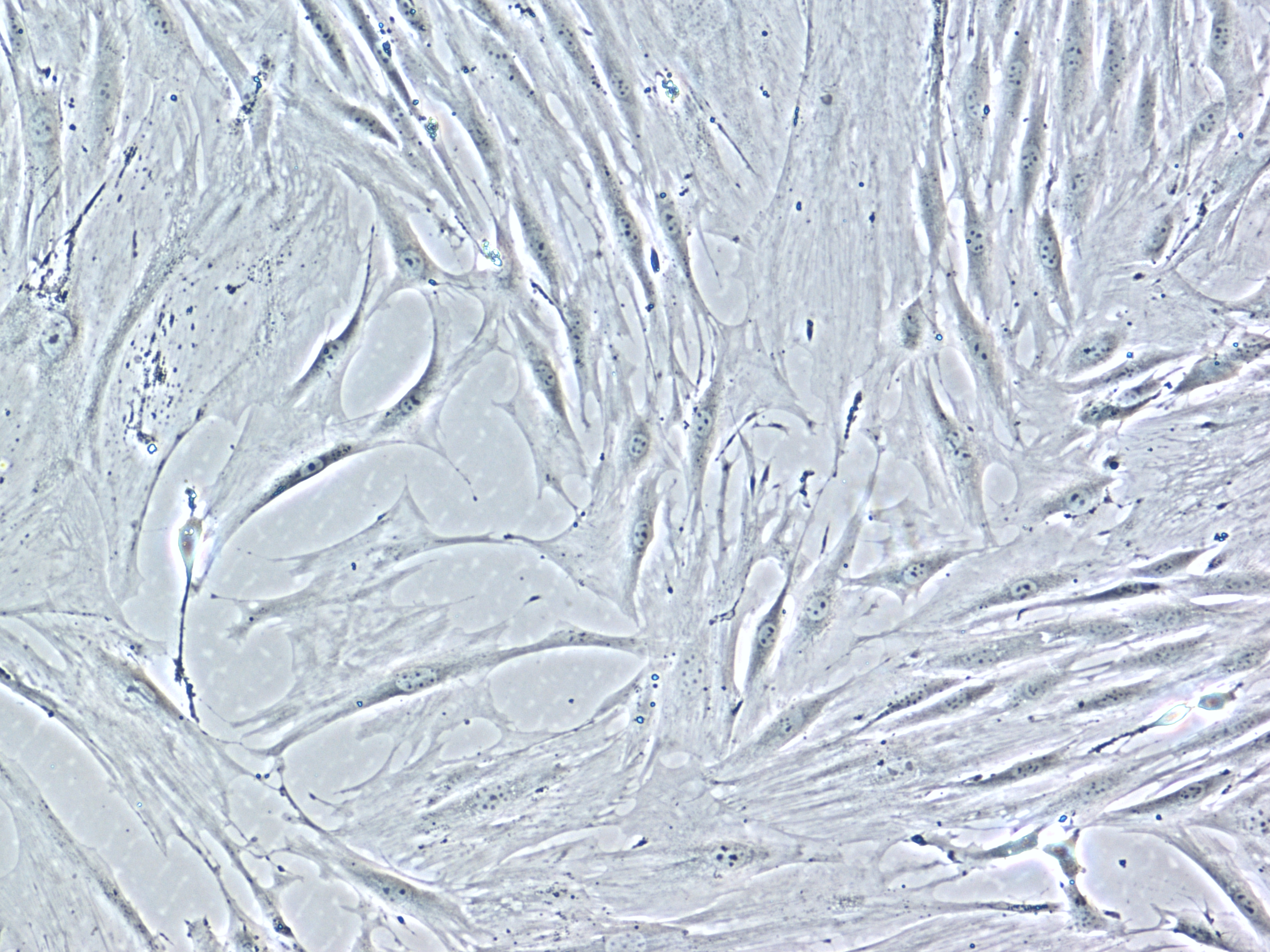

Supplement: Supplementary file 11 — Source data Fig. 7 [file 44318_2025_422_MOESM11_ESM.zip › Figure 7/Fig 7c/Fig 7c_sgWnt9a 2195 vehicle_10X.jpg]

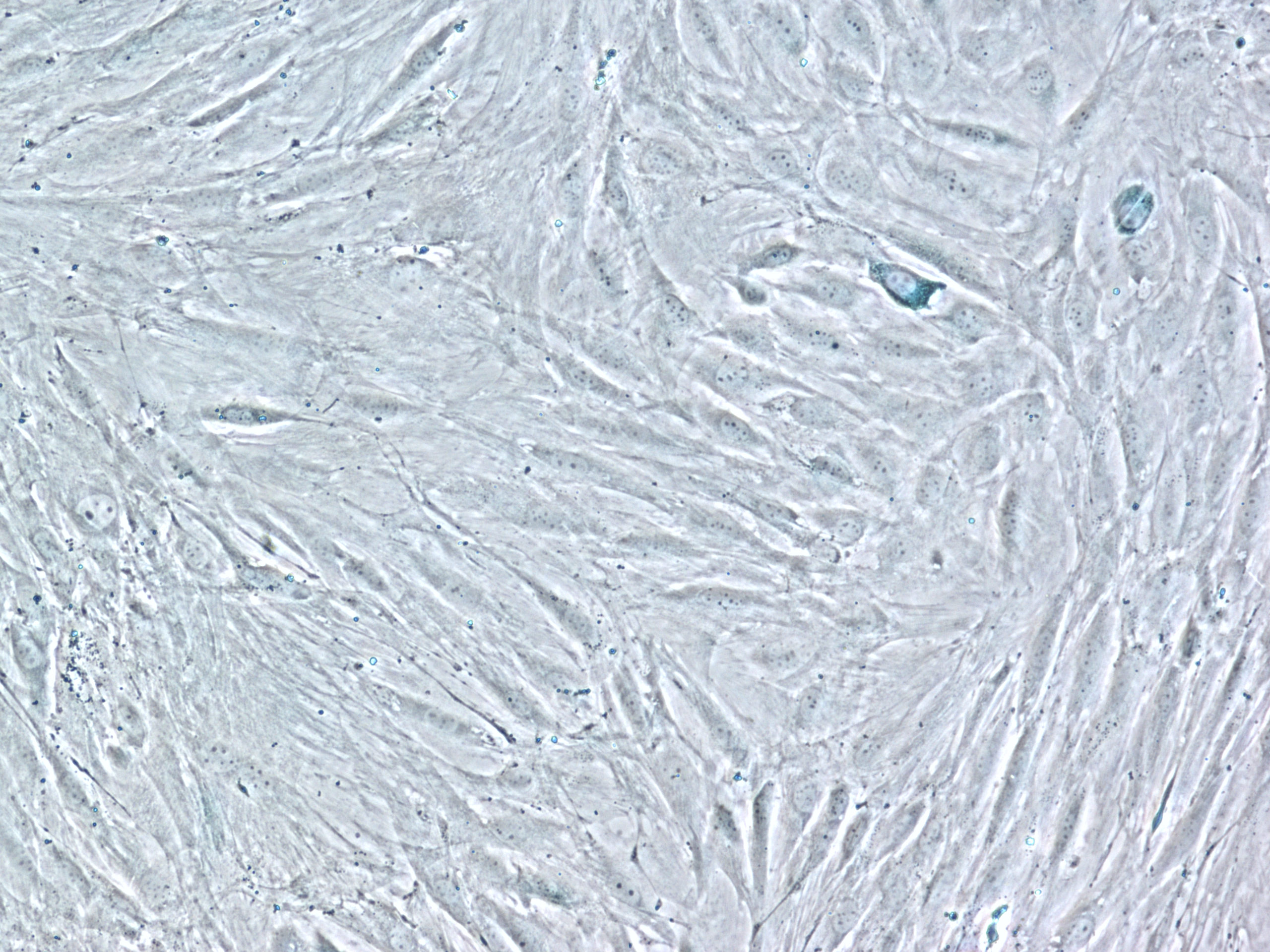

Supplement: Supplementary file 11 — Source data Fig. 7 [file 44318_2025_422_MOESM11_ESM.zip › Figure 7/Fig 7c/Fig 7c_sgNT 1799 vehicle_10X.jpg]

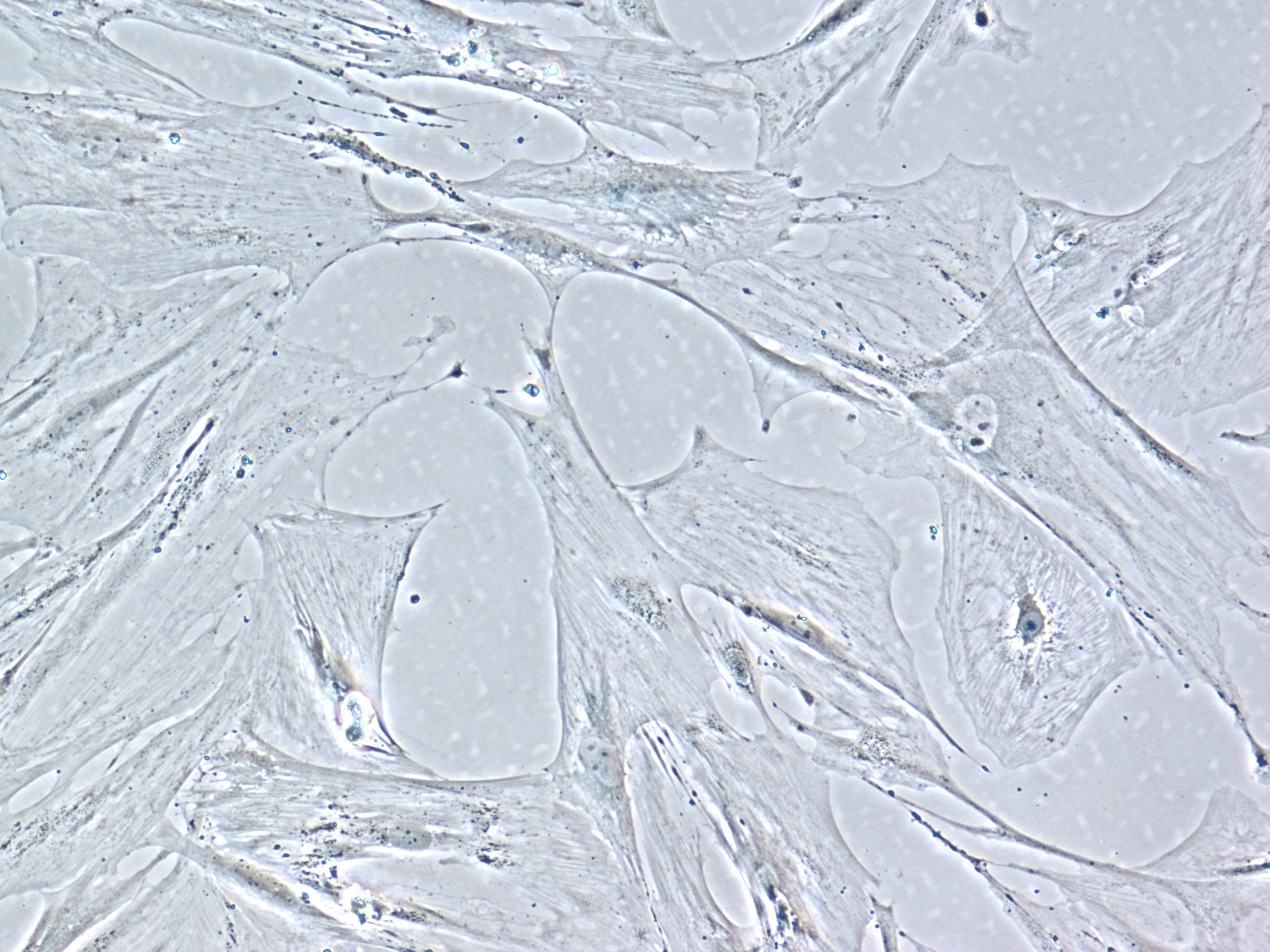

Supplement: Supplementary file 11 — Source data Fig. 7 [file 44318_2025_422_MOESM11_ESM.zip › Figure 7/Fig 7c/Fig 7c_sgWnt9a 2194 etoposide_10X.jpg]

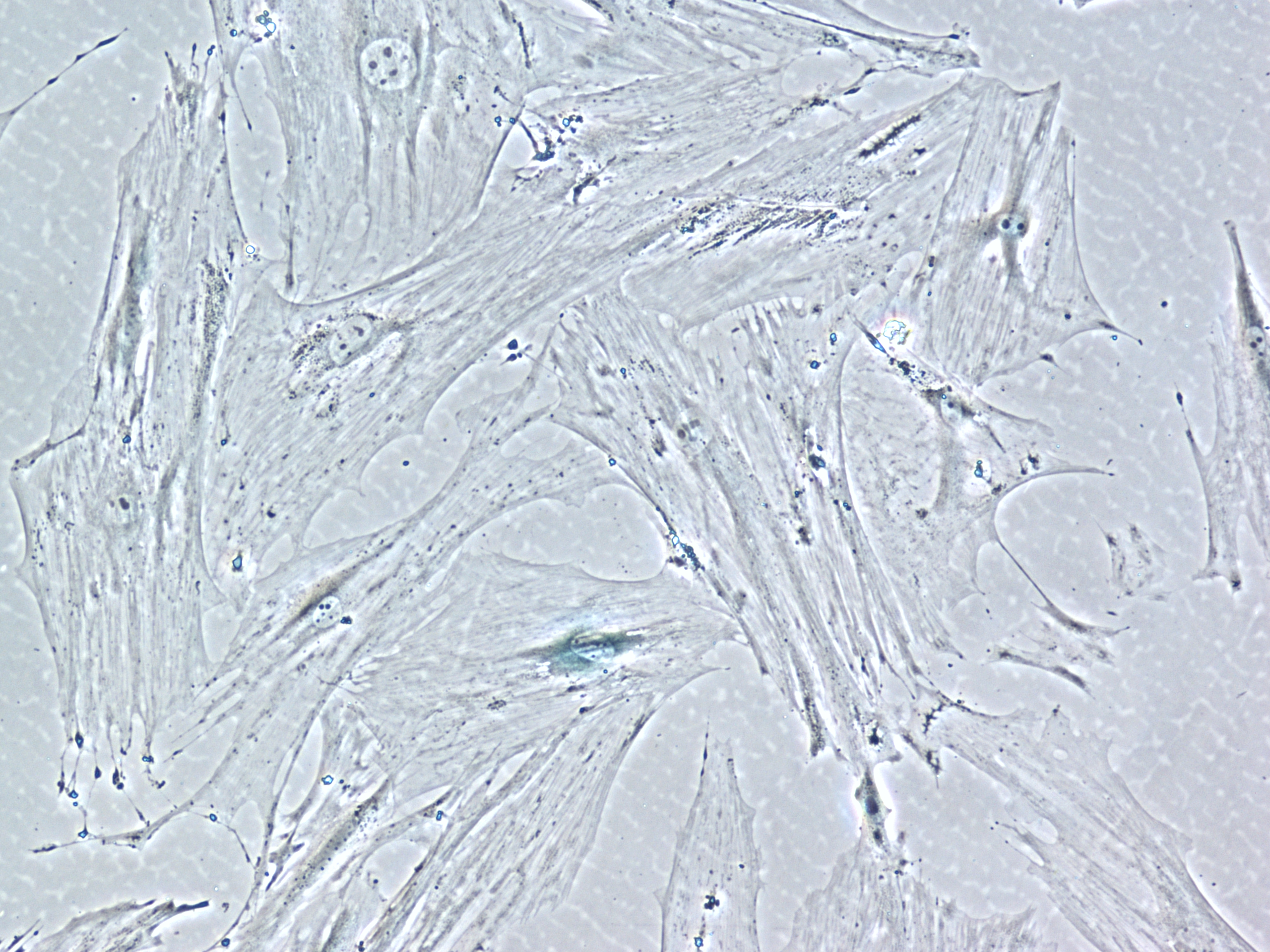

Supplement: Supplementary file 11 — Source data Fig. 7 [file 44318_2025_422_MOESM11_ESM.zip › Figure 7/Fig 7c/Fig 7c_sgWnt9a 2195 etoposide_10X.jpg]

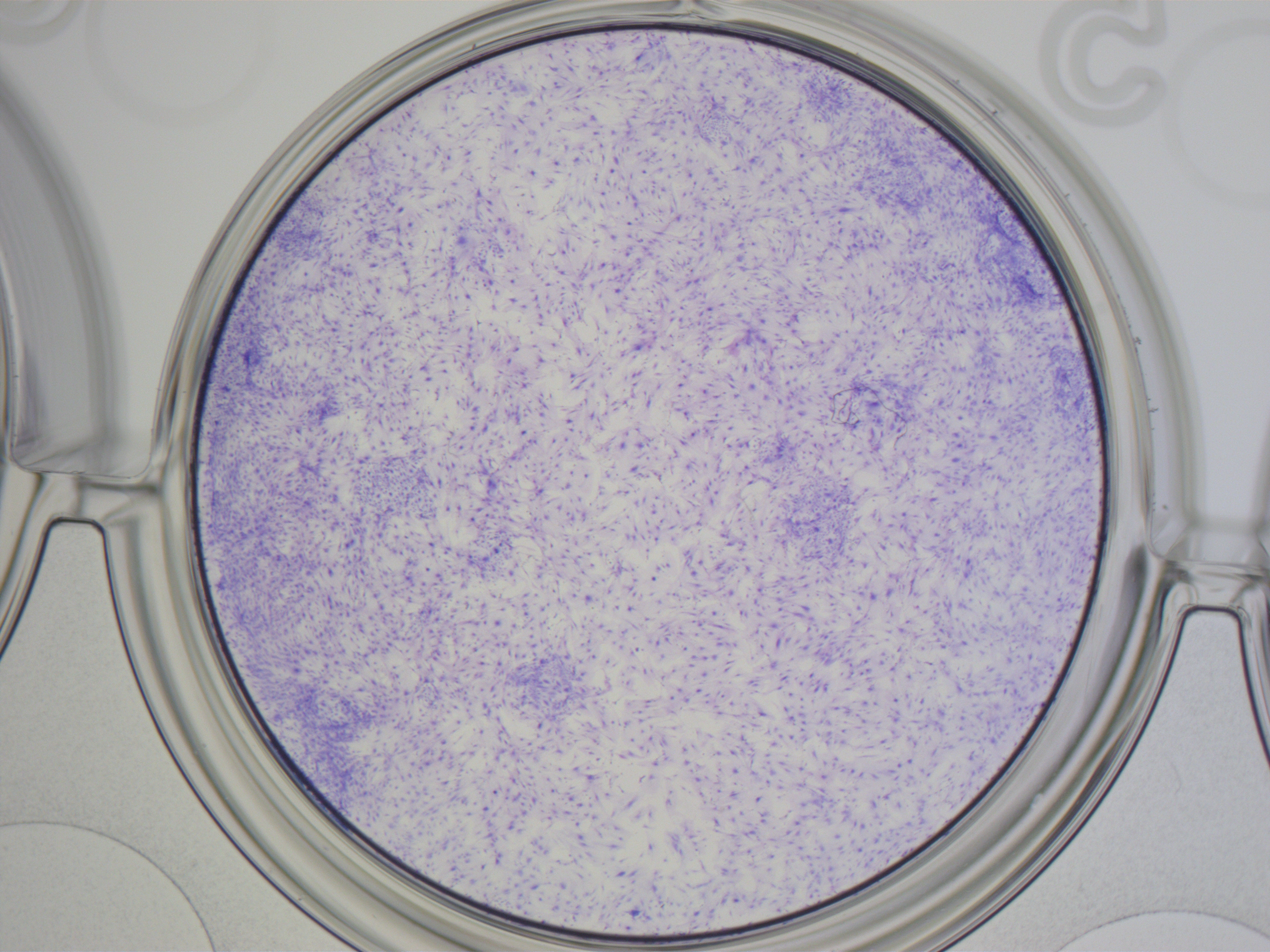

Supplement: Supplementary file 12 — EV Figure Source data [file 44318_2025_422_MOESM12_ESM.zip › Figure EV4/Fig EV4ji/Fig EV4i_GFP+.tiff]

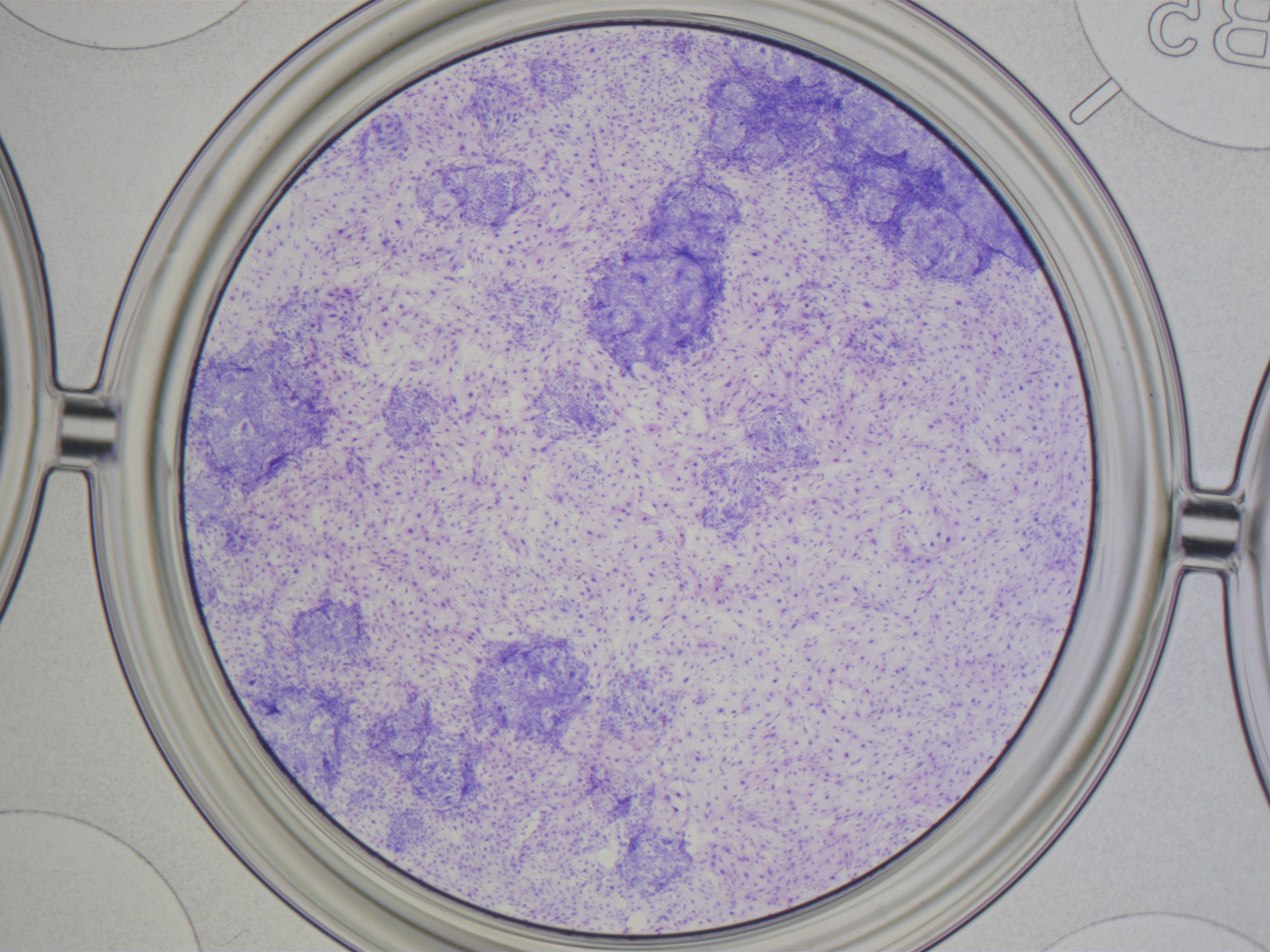

Supplement: Supplementary file 12 — EV Figure Source data [file 44318_2025_422_MOESM12_ESM.zip › Figure EV4/Fig EV4ji/Fig EV4i_GFP-.tiff]

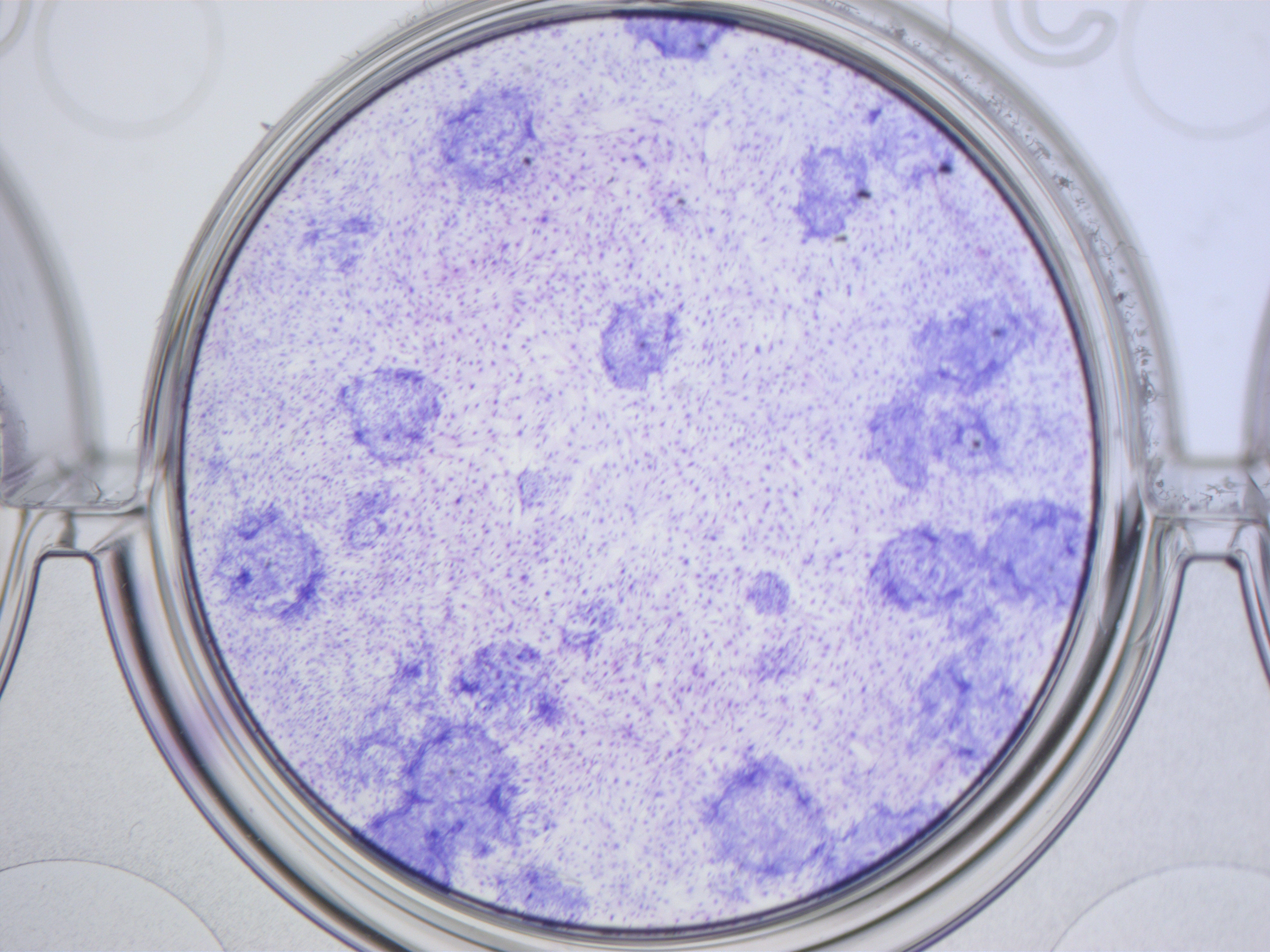

Supplement: Supplementary file 12 — EV Figure Source data [file 44318_2025_422_MOESM12_ESM.zip › Figure EV4/Fig EV4j/Fig EV4j_CD34hi.tiff]

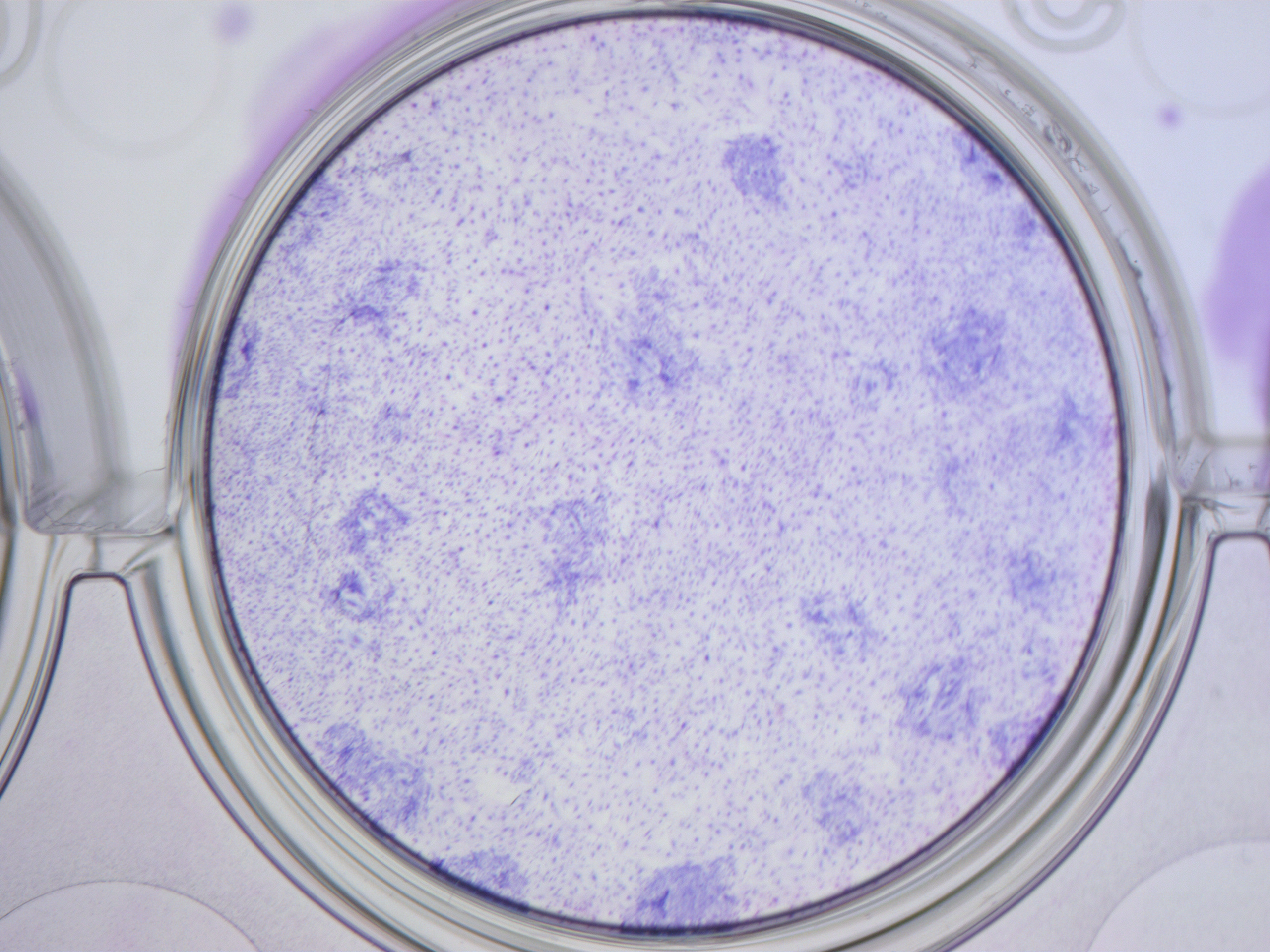

Supplement: Supplementary file 12 — EV Figure Source data [file 44318_2025_422_MOESM12_ESM.zip › Figure EV4/Fig EV4j/Fig EV4j_CD34lo.tiff]
