# Supplementary material for: Fibroblast hierarchy dynamics during mammary gland morphogenesis and tumorigenesis
Source: EMBO J. 2025 Apr 11;44(11):3266–300. doi: 10.1038/s44318-025-00422-3 (PMC12130467; doi:10.1038/s44318-025-00422-3)
Supplement: Supplementary file 13 — Expanded View Figures [file 44318_2025_422_MOESM13_ESM.pdf]

Expanded View Figures

Figure EV1. Normal mammary fibroblast clusters.

(**A**) UMAP plots showing cell types for individual stages before removing contaminant cells (see Table 1). (**B**) UMAP plot of integrated data from all developmental stages colored by *Bmp5* expression. (**C**) Violin plot showing enrichment of the C2 normal signature (lobular-like fibroblasts) in fibroblast clusters found in other tissues (Buechler et al, 2021). Upregulated genes in C2 were used to generate the lobular-like fibroblast signature. (**D**) Bar plot of top KEGG upregulated pathways in one cluster vs the rest. Down- or upregulated genes for each cluster were obtained by pseudo-bulk differential gene expression analysis. (**E**) UMAP plots of integrated data from all developmental stages colored by expression of selected top marker genes for C3. (**F**) Heatmap of gene expression showing the top 15 markers genes for clusters in C1 subclusters (CO^{C1}–$C4^{C1}$). (**G**) UMAP plot for the C1 sub-clustering analysis colored by *Fabp4* expression (**H**) UMAP plot colored by *Dlk1* expression (integrated data for all developmental stages). (**I**) Boxplots for enrichment score of C1 or C2 normal fibroblast transcriptional signatures (Sign.) in human breast tissue with high versus low mammographic density (MD) (Kumar et al, 2023). Box plots show quartiles, minimum and maximum. ***$p < 0.001$, Wilcoxon rank-sum test. (**J**) UMAP plots for an independent integrated scRNA-seq dataset for puberty and adult stages colored by cluster identity (clusters 0–4). (**K**) Dot plot visualization of expression of selected marker genes in each cluster (from Fig. EV1J). The orange square highlights highly expressed genes in cluster 1. The size of the dot encodes the percentage of cells within a cluster, while the color encodes the average expression levels across all cells within a cluster. (**L**) UMAP plots of the sub-clustering of cluster 1 (from Fig. 1EVJ) colored by cluster identity. (**M**) Violin plot for *Crabp1* expression in the cluster 1 subclusters (from Fig. 1EVI).

 © The Author(s)

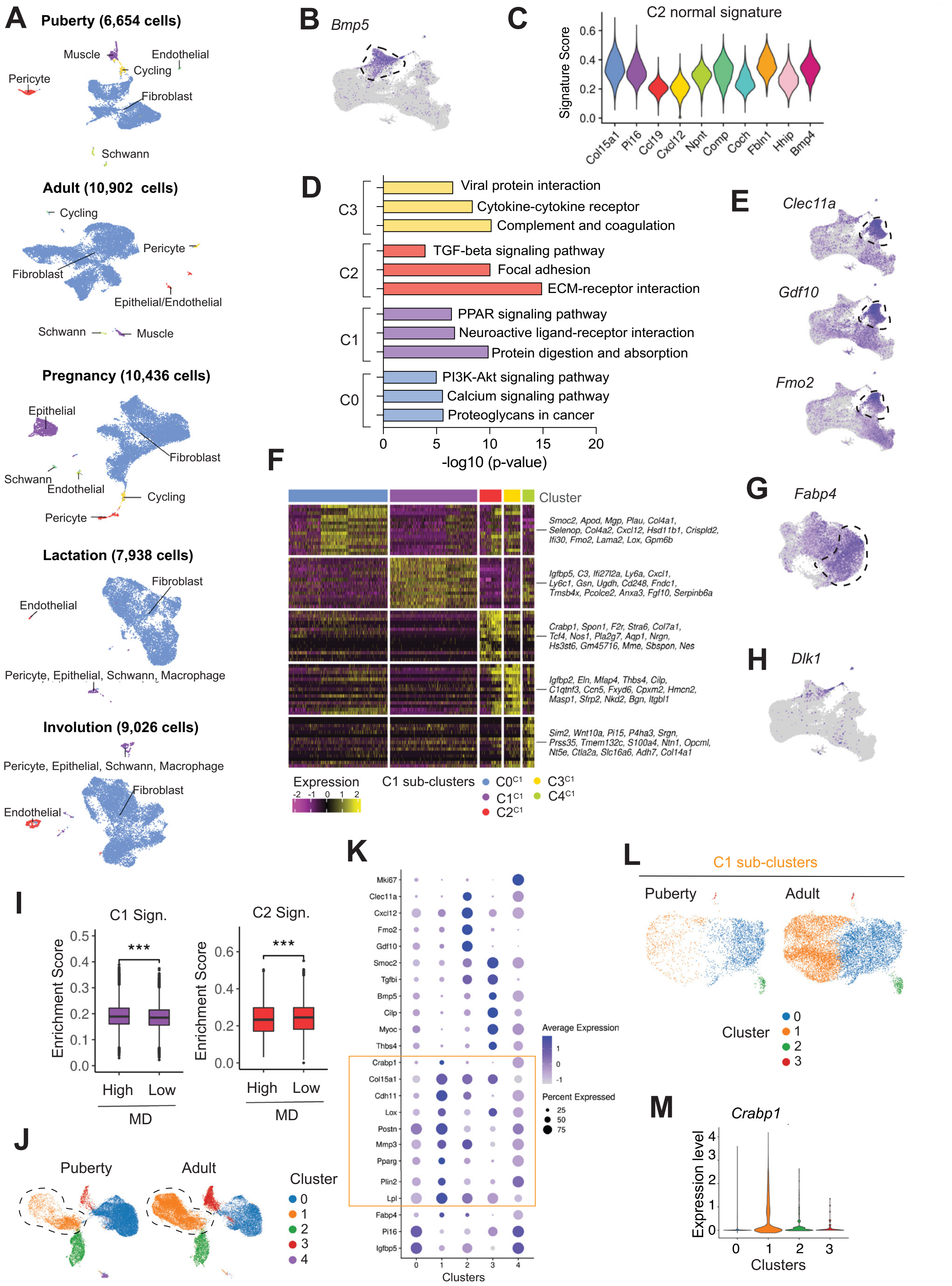
A
Puberty (6,654 cells)
Muscle
Endothelial
Cycling
Pericyte
Fibroblast
Schwann
Adult (10,902 cells)
Cycling
Pericyte
Fibroblast
Epithelial/Endothelial
Schwann
Muscle
Pregnancy (10,436 cells)
Epithelial
Fibroblast
Schwann
Endothelial
Cycling
Pericyte
Lactation (7,938 cells)
Fibroblast
Endothelial
Pericyte, Epithelial, Schwann, Macrophage
Involution (9,026 cells)
Pericyte, Epithelial, Schwann, Macrophage
Fibroblast
Endothelial
B
Bmp5
C
C2 normal signature
Signature Score
Col15a1 Pi16 Ccl19 Cxcl12 Npnt Comp Coch Fbln1 Hhip Bmp4
D
C3
Viral protein interaction
Cytokine-cytokine receptor
Complement and coagulation
C2
TGF-beta signaling pathway
Focal adhesion
ECM-receptor interaction
C1
PPAR signaling pathway
Neuroactive ligand-receptor interaction
Protein digestion and absorption
C0
PI3K-Akt signaling pathway
Calcium signaling pathway
Proteoglycans in cancer
-log10 (p-value)
E
Clec11a
Gdf10
Fmo2
F
Cluster
Smoc2, Apod, Mgp, Plau, Col4a1, Selenop, Col4a2, Cxcl12, Hsd11b1, Crispld2, Ifi30, Fmo2, Lama2, Lox, Gpm6b
Igfbp5, C3, Ifi27l2a, Ly6a, Cxcl1, Ly6c1, Gsn, Ugdh, Cd248, Fndc1, Tmsb4x, Pcolce2, Anxa3, Fgf10, Serpinb6a
Crabp1, Spon1, F2r, Stra6, Col7a1, Tcf4, Nos1, Pla2g7, Aqp1, Nrgn, Hs3st6, Gm45716, Mme, Sbspon, Nes
Igfbp2, Eln, Mfap4, Thbs4, Cilp, C1qtnf3, Ccn5, Fxyd6, Cpxm2, Hmcn2, Masp1, Sfrp2, Nkd2, Bgn, Itgbl1
Sim2, Wnt10a, Pi15, P4ha3, Srgn, Prss35, Tmem132c, S100a4, Ntn1, Opcml, Nt5e, Ctla2a, Slc16a6, Adh7, Col14a1
Expression
C1 sub-clusters
C0C1 C1C1 C2C1 C3C1 C4C1
G
Fabp4
H
Dlk1
I
C1 Sign.
C2 Sign.
Enrichment Score
***
High Low
MD
J
Puberty
Adult
Cluster
K
Mki67 Clec11a Cxcl12 Fmo2 Gdf10 Smoc2 Tgfbi Bmp5 Cilp Myoc Thbs4 Crabp1 Col15a1 Cdh11 Lox Postn Mmp3 Pparg Plin2 Lpl Fabp4 Pi16 Igfbp5
Average Expression
Percent Expressed
Clusters
L
C1 sub-clusters
Puberty
Adult
Cluster
M
Crabp1
Expression level
Clusters

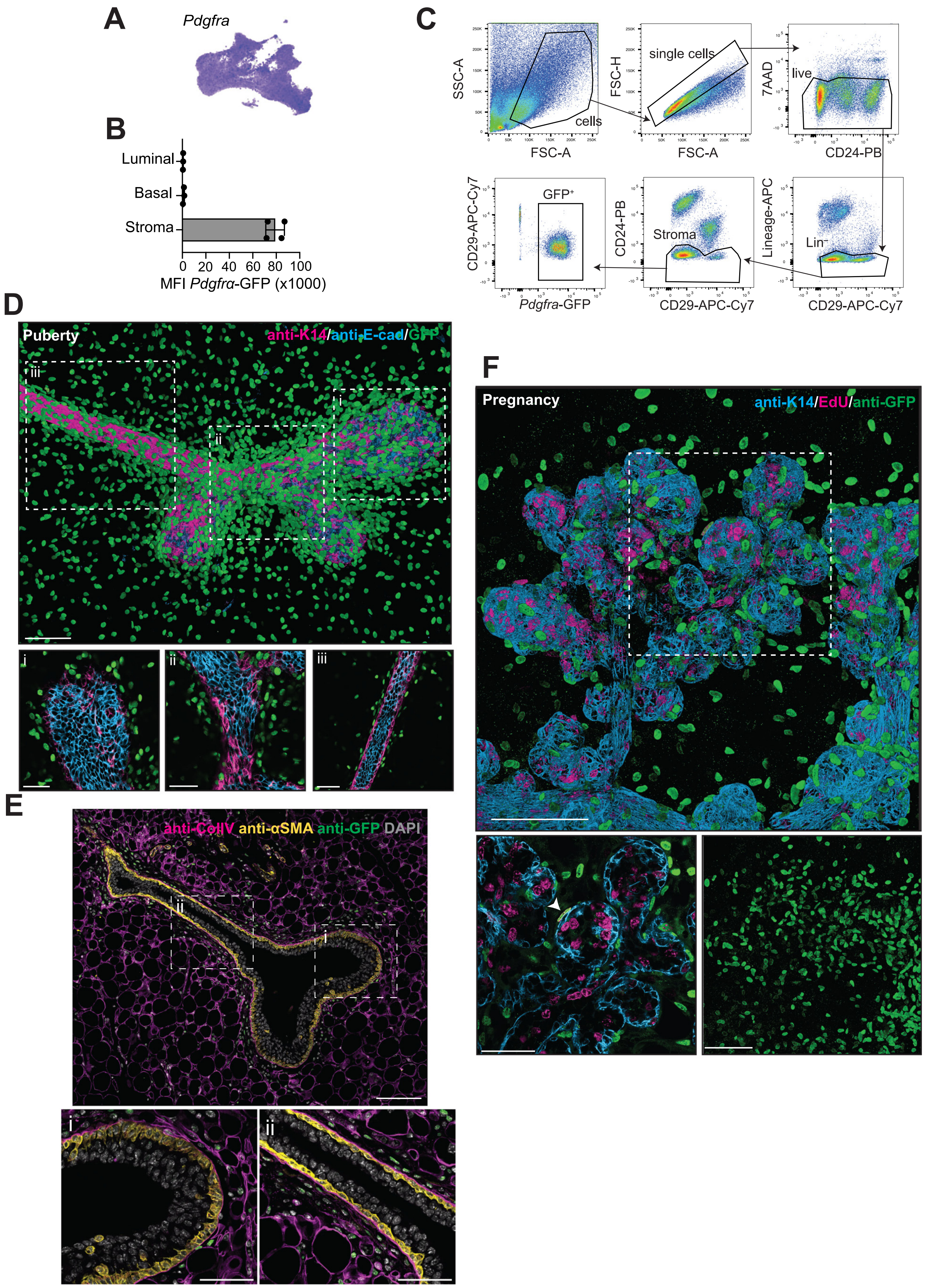
A
Pdgfra
B
Luminal
Basal
Stroma
0 20 40 60 80 100
MFI Pdgfrα-GFP (x1000)
C
SSC-A
FSC-A
cells
FSC-H
single cells
7AAD
live
CD24-PB
CD29-APC-Cy7
GFP+
Pdgfra-GFP
CD24-PB
Stroma
Lineage-APC
Lin−
D
Puberty
anti-K14/anti-E-cad/GFP
i
ii
iii
E
anti-CollV anti-αSMA anti-GFP DAPI
F
Pregnancy
anti-K14/EdU/anti-GFP

Figure EV2. Characterization of fibroblasts using the *Pdgfra*$^{H2B\text{-}GFP}$ model.

(**A**) UMAP plot colored by *Pdgfra* expression. (**B**) Mean fluorescence Intensity (MFI) of *Pdgfra*-GFP levels in the luminal, basal and stromal compartments. Each dot represents an individual mouse ($n = 4$). Error bars, mean ± s.e.m. (**C**) Gating strategy in the *Pdgfra*$^{H2B\text{-}GFP}$ reporter mouse model. (**D**) Representative 3D confocal image and sections of a pubertal *Pdgfra*$^{H2B\text{-}GFP}$ mammary gland (6-week old, $n = 3$). Keratin 14 (K14), E-cadherin and GFP shown in magenta, cyan and green, respectively. Scale bar, 100 µm for wholemounts and 50 µm for optical sections. (**E**) Representative confocal image showing a 6-week-old *Pdgfra*$^{H2B\text{-}GFP}$ mammary gland stained with anti-Collagen IV (ColIV, basement membrane, magenta), anti-α-SMA (myoepithelial, yellow), anti-GFP (fibroblasts, green) and DAPI (gray) ($n = 3$). Scale bar, 100 and 50 µm for zoomed-in images. (**F**) Representative 3D confocal image and optical sections from 12.5-day pregnant *Pdgfra*$^{H2B\text{-}GFP}$ mice injected with EdU 2 h prior to collection ($n = 2$). Keratin 14 (K14), EdU and GFP shown in cyan, magenta and green, respectively. Scale bar, 100 µm for wholemounts and 50 µm for optical sections.

© The Author(s)

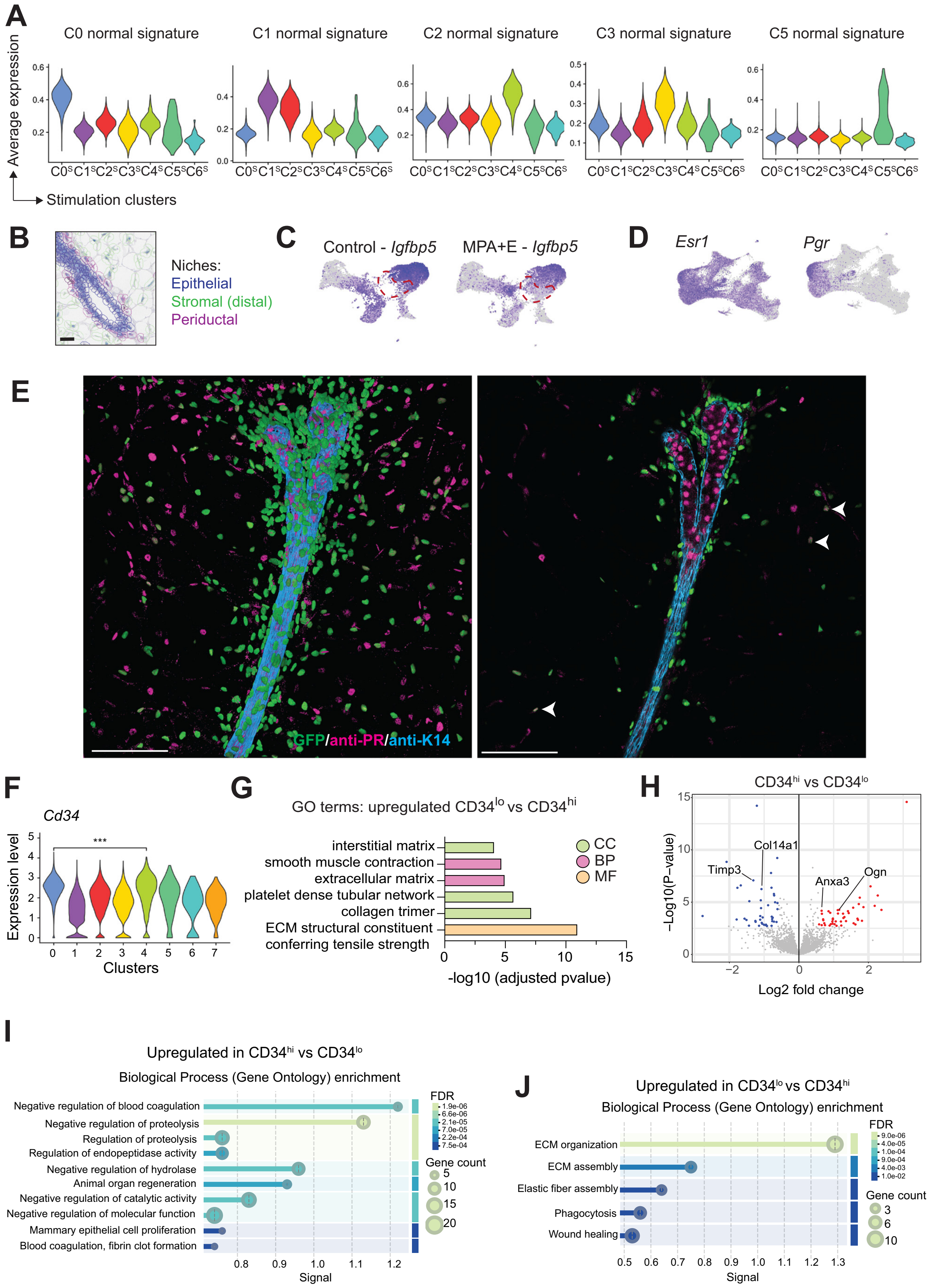
A
C0 normal signature
C1 normal signature
C2 normal signature
C3 normal signature
C5 normal signature
Average expression
Stimulation clusters
B
Niches:
Epithelial
Stromal (distal)
Periductal
C
Control - Igfbp5
MPA+E - Igfbp5
D
Esr1
Pgr
E
GFP/anti-PR/anti-K14
F
Cd34
Expression level
Clusters
G
GO terms: upregulated CD34lo vs CD34hi
interstitial matrix
smooth muscle contraction
extracellular matrix
platelet dense tubular network
collagen trimer
ECM structural constituent conferring tensile strength
CC
BP
MF
-log10 (adjusted pvalue)
H
CD34hi vs CD34lo
Col14a1
Timp3
Anxa3
Ogn
−Log10(P−value)
Log2 fold change
I
Upregulated in CD34hi vs CD34lo
Biological Process (Gene Ontology) enrichment
Negative regulation of blood coagulation
Negative regulation of proteolysis
Regulation of proteolysis
Regulation of endopeptidase activity
Negative regulation of hydrolase
Animal organ regeneration
Negative regulation of catalytic activity
Negative regulation of molecular function
Mammary epithelial cell proliferation
Blood coagulation, fibrin clot formation
FDR
Gene count
Signal
J
Upregulated in CD34lo vs CD34hi
Biological Process (Gene Ontology) enrichment
ECM organization
ECM assembly
Elastic fiber assembly
Phagocytosis
Wound healing
FDR
Gene count
Signal

 © The Author(s)

Figure EV3. Fibroblast subsets during acute hormonal stimulation and characterization of CD34$^{hi/lo}$ fibroblasts.

(**A**) Violin plots showing enrichment of signatures from normal fibroblast clusters in each stimulation cluster (C0^{S}-C6^{S}). Upregulated genes in each normal cluster (post-natal development) were used to generate cluster-specific signatures. (**B**) Snapshot of QuPath classification of mammary niches used in Fig. 3F. Scale bar, 20 µm. (**C**) Separate UMAP plots for control or MPA + E conditions colored by expression of *Igfbp5*. (**D**) UMAP plots of integrated data across post-natal development colored by expression of *Esr1* (left) and *Pgr* (right). (**E**) Representative 3D confocal image and optical section from a *Pdgfra*$^{H2B\text{-}GFP}$ mammary gland showing PR (progesterone receptor, magenta), K14 (Keratin 14, cyan), and GFP (fibroblasts, green) ($n = 2$). Scale bars, 100 µm. Double GFP^{+}PR^{+} cells are highlighted with white arrowheads. (**F**) Violin plots of *Cd34* expression in each cluster. ***$p < 0.001$, Wilcoxon rank-sum test. (**G**) Bar plot of top Gene Ontology (GO) upregulated pathways in CD34^{lo} vs CD34^{hi} fibroblasts by bulk RNA-seq ($n = 3$). (**H**) Volcano plot illustrating the statistical significance ($-\log_{10}$ p value) versus the magnitude of proteomic changes ($\log_2$ fold change) in the secretomes of CD34^{hi} versus CD34^{lo} cultured fibroblasts by mass spectrometry analysis ($n = 3$). Proteins were deemed differentially regulated when the $\log_2$ fold change in protein expression was ≥1-fold and exhibited an adjusted p value ≤0.05. (**I**) Gene ontology pathway analysis of significantly enriched local network clusters (STRING) for proteins upregulated in the CD34^{hi} vs CD34^{lo} secretomes ($n = 3$). For STRING network, active interaction sources include experiments, databases, and co-expression; and minimum required interaction score was 0.700 (medium). FDR, false discovery rate. (**J**) Gene ontology pathway analysis of significantly enriched local network clusters (STRING) for proteins upregulated in CD34^{lo} vs CD34^{hi} fibroblast secretomes ($n = 3$). For STRING network active interaction sources include experiment, databases and co-expression; and minimum required interaction score was 0.700 (medium). FDR, false discovery rate.

© The Author(s)

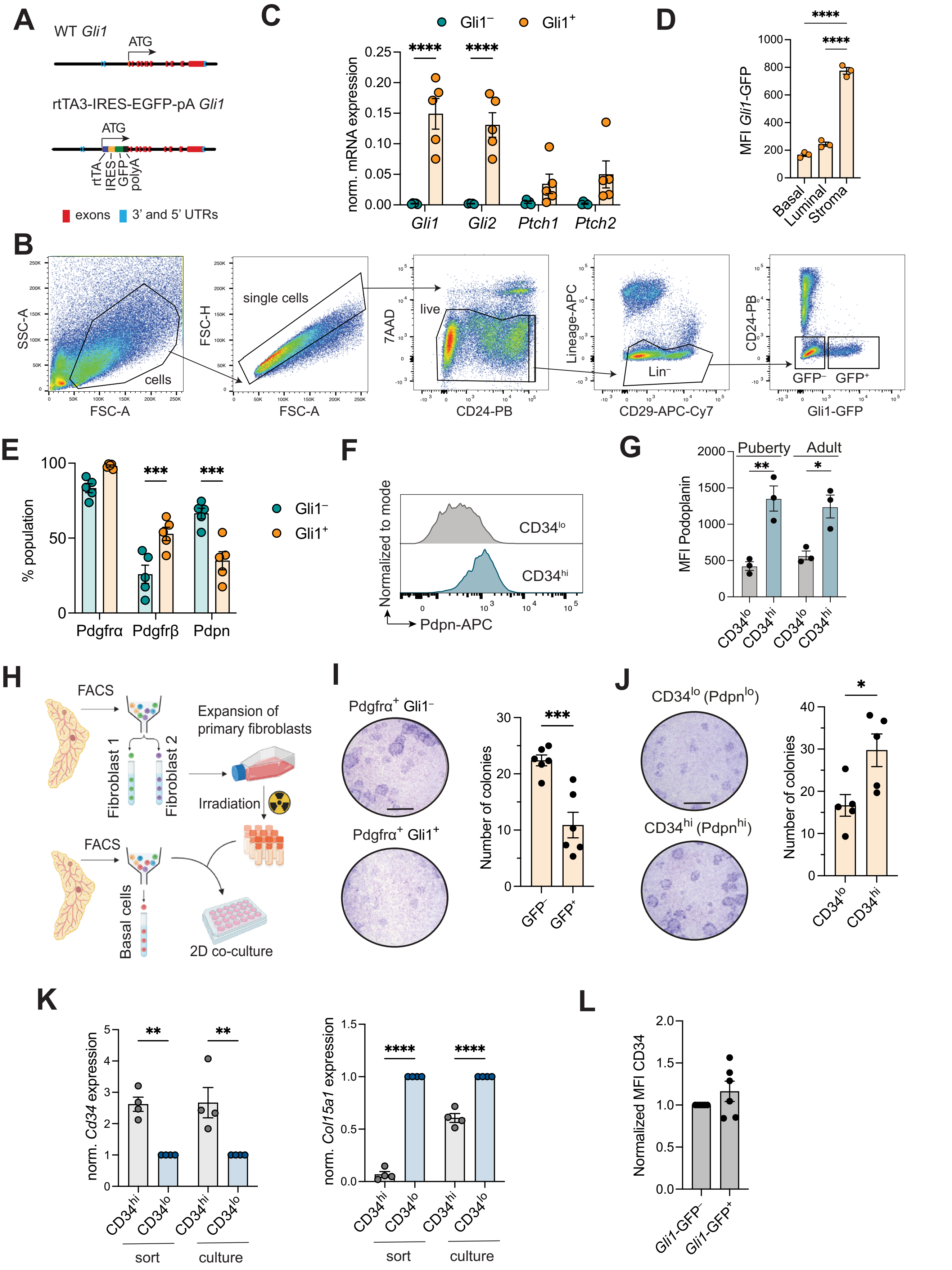
A
WT Gli1
ATG
rtTA3-IRES-EGFP-pA Gli1
ATG
rtTA
IRES
GFP
polyA
exons
3' and 5' UTRs
C
Gli1⁻
Gli1⁺
norm. mRNA expression
****
****
Gli1
Gli2
Ptch1
Ptch2
D
****
****
MFI Gli1-GFP
Basal
Luminal
Stroma
B
SSC-A
FSC-A
cells
FSC-H
single cells
7AAD
live
CD24-PB
Lineage-APC
Lin⁻
CD29-APC-Cy7
CD24-PB
GFP⁻
GFP⁺
Gli1-GFP
E
% population
***
***
Pdgfrα
Pdgfrβ
Pdpn
Gli1⁻
Gli1⁺
F
Normalized to mode
CD34lo
CD34hi
Pdpn-APC
G
Puberty
Adult
**
*
MFI Podoplanin
CD34lo
CD34hi
CD34lo
CD34hi
H
FACS
Fibroblast 1
Fibroblast 2
Expansion of
primary fibroblasts
Irradiation
FACS
Basal cells
2D co-culture
I
Pdgfrα⁺ Gli1⁻
Pdgfrα⁺ Gli1⁺
***
Number of colonies
GFP⁻
GFP⁺
J
CD34lo (Pdpnlo)
CD34hi (Pdpnhi)
*
Number of colonies
CD34lo
CD34hi
K
norm. Cd34 expression
**
**
CD34hi
CD34lo
CD34hi
CD34lo
sort
culture
norm. Col15a1 expression
****
****
CD34hi
CD34lo
CD34hi
CD34lo
sort
culture
L
Normalized MFI CD34
Gli1-GFP⁻
Gli1-GFP⁺

 © The Author(s)

Figure EV4. Characterization of Gli1^{+} specialized fibroblasts.

(**A**) Targeting strategy to generate Gli1 rtTA3-IRES-EGFP-pA reporter mice. WT, wild-type. rtTA, reverse tetracycline-controlled trans-activator. IRES, internal ribosome entry sites. UTR, untranslated region. (**B**) Gating strategy for the Gli1-rtTA-IRES-GFP KI mouse model ($n = 8$). (**C**) mRNA expression of *Gli1*, *Gli2*, *Ptch1*, and *Ptch2* normalized to *Gapdh* expression in *Gli1*-GFP^{-} and *Gli1*-GFP^{+} mammary fibroblasts sorted from adult female mice. Each dot represents an individual mouse ($n = 5$). Error bars, mean ± s.e.m., $****p < 0.0001$, two-way ANOVA. (**D**) Mean Fluorescence Intensity (MFI) of *Gli1*-GFP expression in basal (CD29^{hi} CD24^{lo}), luminal (CD29^{lo} CD24^{hi}) and stromal (CD24^{-}) populations. Each dot represents an individual mouse ($n = 3$). Error bars, mean ± s.e.m., $****p < 0.0001$, ordinary one-way ANOVA. (**E**) Percentage of Pdgfrα-, Pdgfrβ- or Pdpn (Podoplanin)-positive cells in *Gli1*-GFP$^{+/-}$ stroma assessed by flow cytometry. Each dot represents an individual mouse ($n = 5$). Error bars, mean ± s.e.m., $***p < 0.001$, two-way ANOVA. (**F**) Representative histograms for Pdpn expression in Pdgfrα^{+}CD34$^{hi/lo}$ fibroblasts from 6-week-old FVB/NJ mice by flow cytometry ($n = 3$). (**G**) Bar plot of MFI for Pdpn levels in Pdgfrα^{+}CD34$^{hi/lo}$ populations in pubertal (6-week old, $n = 3$) and adult (9-week old, $n = 3$) FVB/NJ mice. Error bars, mean ± s.e.m., $*p < 0.05$, $**P < 0.01$, unpaired *t*-test. MFI mean fluorescence intensity. (**H**) Workflow for 2D co-culture assays with primary irradiated fibroblasts and freshly sorted basal epithelial cells. Created with BioRender.com. (**I**) Representative images (left) and quantification (right) of basal colonies seeded with Pdgfrα^{+} *Gli1*-GFP^{+} or Pdgfrα^{+} *Gli1*-GFP^{-} cells ($n = 6$). Each dot represents the average of three replicates, each condition includes two independent sets of fibroblasts. Error bars, mean ± s.e.m., $***p < 0.001$, unpaired *t*-test. Scale bar, 5 mm. (**J**) Representative images (left) and quantification (right) of basal/myoepithelial colonies seeded with Pdgfrα^{+}CD34$^{hi/lo}$ fibroblasts ($n = 5$). Each dot represents the average of three replicates, each condition includes two independent sets of fibroblasts. Error bars, mean ± s.e.m., $*p < 0.05$, unpaired *t*-test. Scale bar, 5 mm. (**K**) mRNA expression of *Cd34* (left) or *Col1a51a1* (right) genes normalized to *Gapdh* expression (housekeeping gene) in CD34^{hi} and CD34^{lo} mammary fibroblasts freshly sorted or after one passage in culture. Each expression value for CD34^{hi} cells was normalized to their CD34^{lo} counterpart. Each dot represents an individual mouse ($n = 4$). Error bars, mean ± s.e.m., $**p < 0.01$, $****p < 0.0001$, ordinary one-way ANOVA. (**L**) Mean fluorescence intensity (MFI) of CD34 expression in *Gli1*-GFP$^{+/-}$ CAFs from Gli1-Wnt1 tumors, normalized by the *Gli1*-GFP^{-} population for each tumor. Each dot represents a tumor ($n = 6$). Error bars, mean ± s.e.m. Source data are available online for this figure.

© The Author(s)

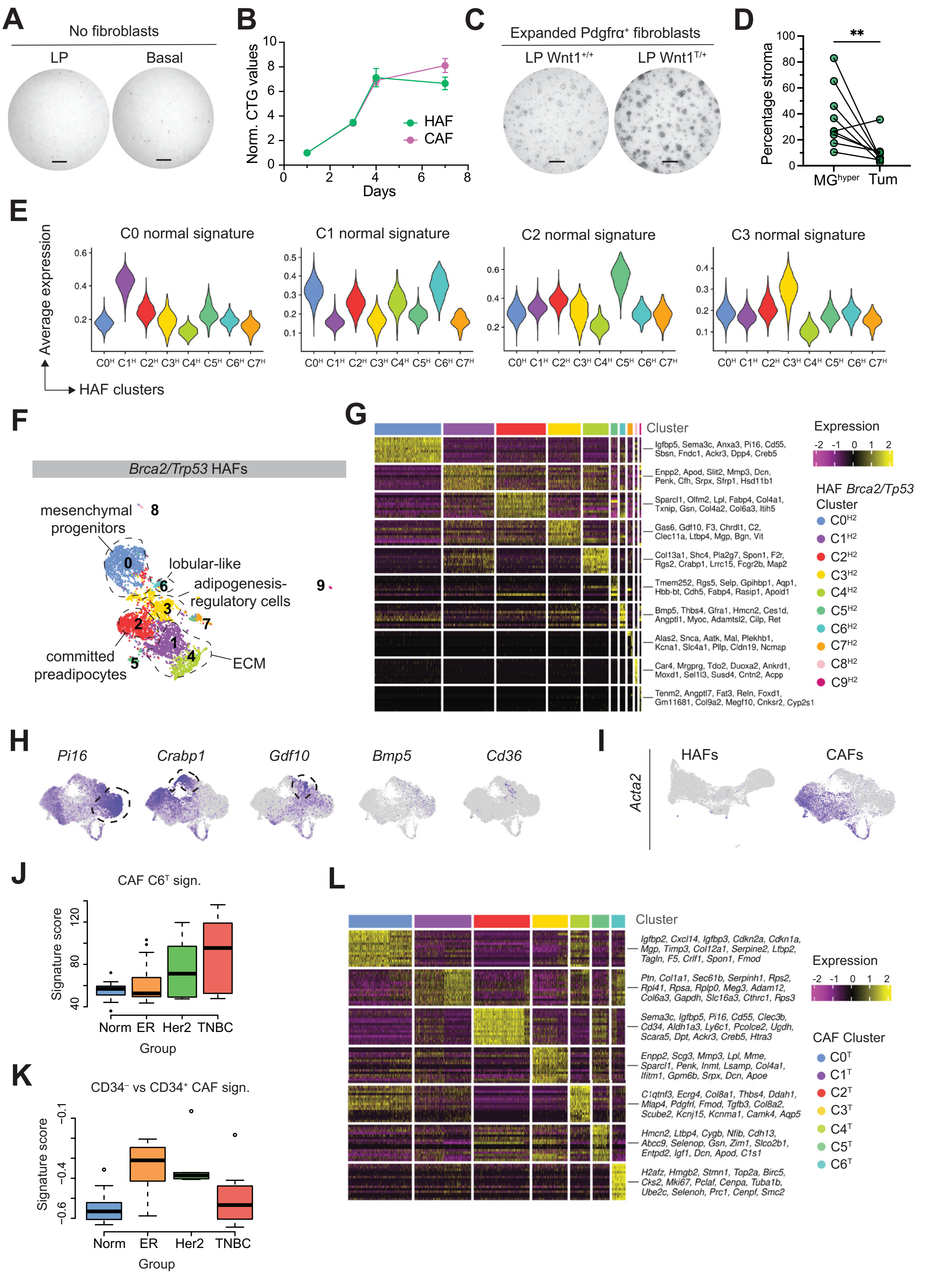
A
No fibroblasts
LP
Basal
B
Norm. CTG values
Days
HAF
CAF
C
Expanded Pdgfrα+ fibroblasts
LP Wnt1+/+
LP Wnt1T/+
D
Percentage stroma
**
MGhyper
Tum
E
Average expression
C0 normal signature
C1 normal signature
C2 normal signature
C3 normal signature
HAF clusters
F
Brca2/Trp53 HAFs
mesenchymal progenitors
lobular-like
adipogenesis-regulatory cells
committed preadipocytes
ECM
G
Cluster
Expression
HAF Brca2/Tp53 Cluster
H
Pi16
Crabp1
Gdf10
Bmp5
Cd36
I
Acta2
HAFs
CAFs
J
CAF C6T sign.
Signature score
Norm
ER
Her2
TNBC
Group
K
CD34− vs CD34+ CAF sign.
L
Cluster
Expression
CAF Cluster

	© The Author(s)

Figure EV5. Characterization of hyperplasia- and cancer-associated fibroblasts.

(**A**) Representative images of 3D colony-forming assays with basal and luminal progenitor (LP) cells without fibroblasts. Scale bars, 200 µm. (**B**) CellTiter-Glo (CTG) values, normalized to day 1, were used to assess the growth of HAFs and CAFs. $n = 3$ sets of matched raGFP-Wnt1 HAFs and CAFs. Error bars, mean ± s.e.m. (**C**) Representative images of 3D colony-forming assays with luminal progenitor (LP) cells from hyperplastic glands (16-week-old FVB-Wnt1$^{T/+}$) or littermate controls (FVB-Wnt1$^{+/+}$) co-cultured with Pdgfrα^{+} fibroblasts. Scale bars, 200 µm. (**D**) Quantification of the percentage of stroma (Lin^{-}CD24^{-}) in raGFP-Wnt1 hyperplastic mammary glands and tumors. Each dot represents an individual mouse ($n = 9$). Error bars, mean ± s.e.m., $**p < 0.01$, unpaired *t*-test. (**E**) Violin plots for the enrichment of signatures from normal fibroblast clusters in each raGFP-Wnt1 HAF cluster (C0^{H}-C7^{H}). Upregulated genes in each normal cluster were used to generate cluster-specific signatures. HAF clusters are indicated by color. (**F**) UMAP plot of HAFs in *Brca2/Trp53*-deficient hyperplastic tissue showing ten clusters (C0^{H2}-C9^{H2}) indicated by color. Clusters were annotated according to their expression profiles. (**G**) Heatmap of gene expression for the top 10 marker genes for each HAF cluster in *Brca2/Trp53*-deficient glands (C0^{H2}-C9^{H2}). (**H**) UMAP plot (raGFP-Wnt1 CAFs) colored by expression of selected marker genes. (**I**) UMAP plots (raGFP-Wnt1 HAF and CAFs) colored by expression of *Acta2*. (**J**) Box plots showing enrichment of the mouse C6^{T} CAF cluster transcriptional signature in normal human fibroblasts ($n = 13$) or CAFs in different breast cancer subtypes. ER, estrogen receptor ($n = 13$). HER2, human epidermal growth factor receptor 2 ($n = 6$). TNBC, triple-negative breast cancer ($n = 8$). Box plots show quartiles, minimum and maximum. (**K**) Box plots showing enrichment of the raGFP-Wnt1 CD34^{-} CAF signature generated by bulk differential gene expression analysis ($n = 3$) in normal human fibroblasts or CAFs in different breast cancer subtypes. Box plots show quartiles, minimum and maximum. (**L**) Heatmap showing expression of the top 15 marker genes for each cluster in raGFP-Wnt1 CAFs (C0^{T}–C6^{T}).

© The Author(s)

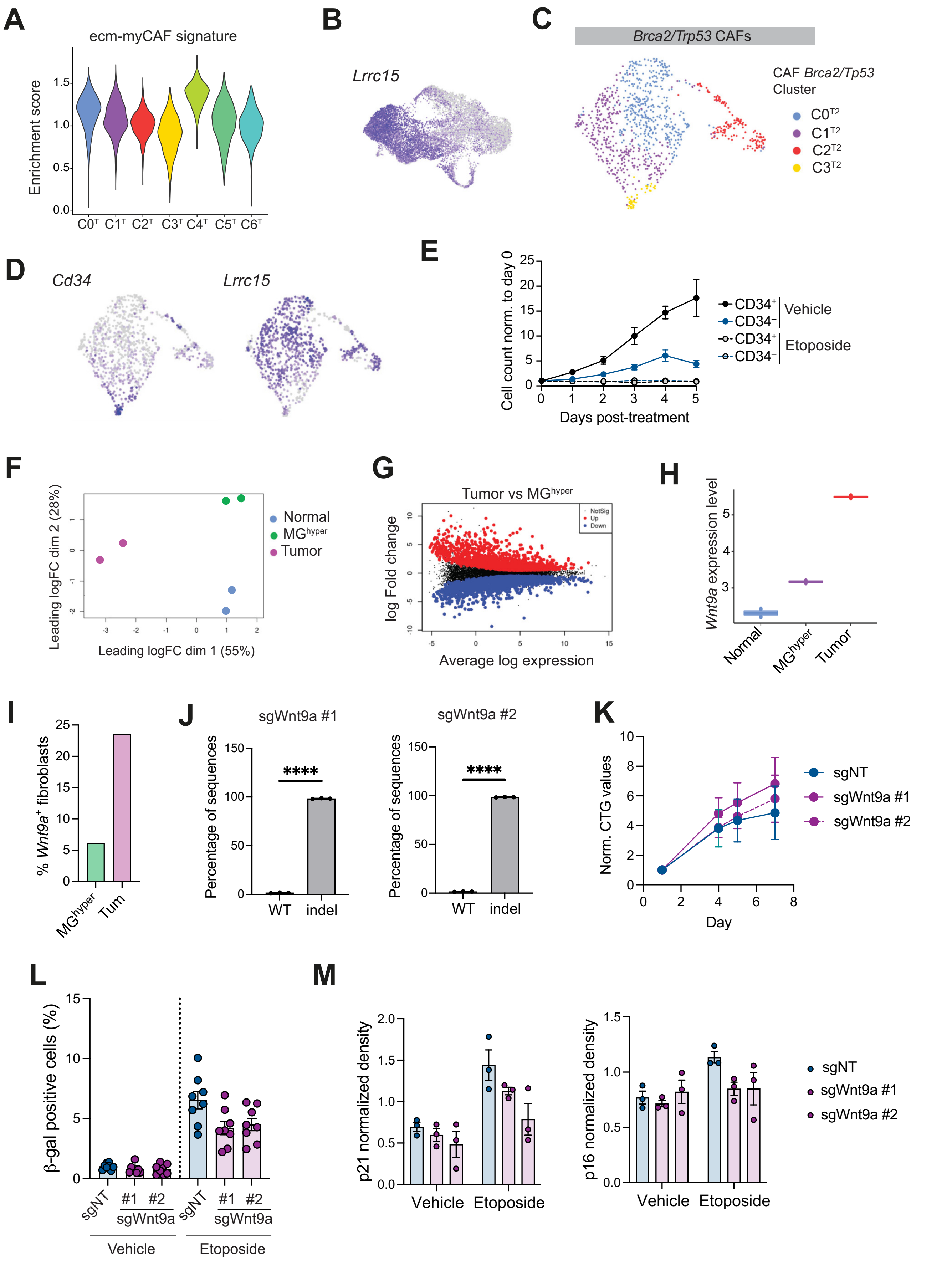
A
ecm-myCAF signature
Enrichment score
C0T C1T C2T C3T C4T C5T C6T
B
Lrrc15
C
Brca2/Trp53 CAFs
CAF Brca2/Tp53 Cluster
C0T2
C1T2
C2T2
C3T2
D
Cd34
Lrrc15
E
Cell count norm. to day 0
Days post-treatment
CD34+
CD34–
Vehicle
CD34+
CD34–
Etoposide
F
Leading logFC dim 2 (28%)
Leading logFC dim 1 (55%)
Normal
MGhyper
Tumor
G
Tumor vs MGhyper
log Fold change
Average log expression
NotSig
Up
Down
H
Wnt9a expression level
Normal
MGhyper
Tumor
I
% Wnt9a+ fibroblasts
MGhyper
Tum
J
sgWnt9a #1
sgWnt9a #2
Percentage of sequences
****
WT
indel
K
Norm. CTG values
Day
sgNT
sgWnt9a #1
sgWnt9a #2
L
β-gal positive cells (%)
sgNT
#1 #2
sgWnt9a
Vehicle
Etoposide
M
p21 normalized density
p16 normalized density
Vehicle
Etoposide
sgNT
sgWnt9a #1
sgWnt9a #2

 © The Author(s)

Figure EV6. Molecular features of CD34⁻ myCAFs.

(**A**) Violin plots for enrichment of the ecm-myCAF signature (Kieffer et al, 2020) in each tumor cluster ($C0^T$–$C6^T$). (**B**) UMAP plot (raGFP-Wnt1 CAFs) colored by *Lrrc15* expression. (**C**) UMAP plot (CAFs, *Brca2/Trp53*-deficient model) showing four clusters ($C0^{T2}$–$C3^{T2}$) indicated by color. (**D**) UMAP plot (CAFs, *Brca2/Trp53*-deficient model) colored by expression of *Cd34* (left) and *Lrrc15* (right). (**E**) Cell counts for $CD34^+$ or $CD34^-$ raGFP-Wnt1 CAFs treated with vehicle or etoposide normalized to day 0 ($n = 4$, two independent sets of primary fibroblasts). Error bars, mean ± s.e.m. (**F**) Multidimensional scaling (MDS) plot for pseudo-bulk gene expression analysis of all fibroblasts in normal tissue (puberty + adult), hyperplastic tissue (MG^{hyper}, $n = 2$), and tumors ($n = 2$). (**G**) Mean-difference plot showing differentially expressed genes between fibroblasts in tumors vs hyperplastic glands MG^{hyper} ($n = 2$). Significantly upregulated and downregulated genes are shown as red and blue dots, respectively. (**H**) *Wnt9a* expression in pseudo-bulk data for normal, hyperplastic (raGFP-Wnt1 MG^{hyper}, $n = 2$) and raGFP-Wnt1 tumor states ($n = 2$). (**I**) Percentage of *Wnt9a*⁺ fibroblasts in hyperplastic tissue and tumors in *Brca2/Trp53*-deficient mice assessed by scRNA-seq ($n = 2$ mice per timepoint). (**J**) Percentage of WT (wild-type) or mutated (indels) sequences in $CD34^-$ myCAFs transduced with sgRNAs targeting the *Wnt9a* locus (sgWnt9a). Each dot represents a replicate ($n = 2$). ****$p < 0.0001$, unpaired *t*-test. Error bars, mean ± s.e.m. (**K**) CellTiter-Glo (CTG) values, normalized to day 1, to assess growth of control (sgNT) and Wnt9a-KO $CD34^-$ myCAFs. $n = 2$ independent sets of CRISPR-edited $CD34^-$ myCAFs performed in triplicate. Error bars, mean ± s.e.m. (**L**) Quantification of the percentage of β-galactosidase⁺ $CD34^-$ myCAFs, either Wnt9a-KO or control (sgNT) cells treated with vehicle or etoposide. Values were normalized to the average of the untreated control for each experiment. Raw values for the etoposide-treated conditions are the same as in Fig. 6I. Each dot represents a technical duplicate, $n = 4$ independent experiments with two independent sets of primary CAFs. Error bars, mean ± s.e.m. (**M**) Quantification of p21 (left) and p16 (right) protein band density, normalized to Vinculin, evaluated by western blot analysis of control (sgNT) or Wnt9a-KO raGFP-Wnt1 $CD34^-$ myCAFs treated with vehicle or etoposide ($n = 3$). Error bars, mean ± s.e.m.

© The Author(s)
